# Integrated Transcriptomics, Proteomics, and Glycomics Reveals the Association between Up-regulation of Sialylated N-glycans/Integrin and Breast Cancer Brain Metastasis

**DOI:** 10.1038/s41598-019-53984-8

**Published:** 2019-11-22

**Authors:** Wenjing Peng, Rui zhu, Shiyue Zhou, Parvin Mirzaei, Yehia Mechref

**Affiliations:** Department of Chemistry and Biochemistry, Texas Tech University, Lubbock, TX USA

**Keywords:** Protein-protein interaction networks, Breast cancer

## Abstract

Breast cancer brain metastasis has been recognized as one of the central issues in breast cancer research. The elucidation of the processes and pathways that mediate this step will provide important clues for a better understanding of breast cancer metastasis. Increasing evidence suggests that aberrant glycosylation patterns greatly contribute to cell invasion and cancer metastasis. Herein, we combined next-generation RNA sequencing with liquid chromatography-tandem mass spectrometry-based proteomic and N-glycomic analysis from five breast cancer cell lines and one brain cancer cell line to investigate the possible mechanisms of breast cancer brain metastasis. The genes/proteins associated with cell movement were highlighted in breast cancer brain metastasis. The integrin signaling pathway and the up-regulation of α-integrin (ITGA2, ITGA3) were associated with the brain metastatic process. 12 glycogenes showed unique expression in 231BR, which could result in an increase of sialylation during brain metastasis. In agreement with the changes of glycogenes, 60 out of 63 N-glycans that were identified exhibited differential expression among cell lines. The correlation between glycogenes and glycans revealed the importance of sialylation and sialylated glycans in breast cancer brain metastasis. Highly sialylated N-glycans, which were up-regulated in brain-seeking cell line 231BR, likely play a role in brain metastasis.

## Introduction

Breast cancer, the leading cause of cancer mortality and morbidity among females, represents the second highest frequency of brain metastasis^1,2^. The incidence of brain metastasis among breast cancer patients is continuously increasing due to the development of detection technologies for better prognosis and the improvement of therapeutic methods leading to a longer survival of primary breast cancer patients^3^. Thus, breast cancer brain metastasis is considered an essential issue in breast cancer studies^4–8^.

The key event of breast cancer brain metastasis is the penetration of breast cancer cells through the blood-brain barrier^9^. The blood-brain barrier acts as a selective “physical barrier” around the brain, due to the tight junction and adherens junction between adjacent brain microvascular endothelial cells (BMECs)^4,10^. BMECs provide low permeability, high electrical resistance, and a series of transport channels that supply the brain with nutrients and mediate the waste products of brain metabolism, so that the blood-brain barrier can protect the brain from toxins, unnecessary proteins, and cells^4,11^. However, breast cancer cells can penetrate this defensive layer by attaching to and invading the blood-brain barrier. In addition to protecting the brain, the blood-brain barrier can also prevent drugs from entering the brain; consequently, brain metastasis treatments pose a great challenge. Current systematic therapy, including chemotherapy, hormone therapy, and targeted-drug therapy, is not effective when treating breast cancer brain metastatic sites^12^. Thus, in recent years breast cancer brain metastasis has attracted much research interest, and many efforts have been made to elucidate the mechanisms of this process^6,7,12–14^. Overexpression of HER2 is considered to be associated with breast cancer brain metastasis and can be used as a positive prognostic factor^15–17^. Parul Gupta *et al*. reported that the suppression of HER2, EGFR, and VEGF may suppress breast cancer brain metastasis *in vivo*^6^. The methylation of GALNT9, CCDC8, and BNC1 genes have also been investigated in brain metastasis^18^. In 2009, Massague and coworkers reported that COX2 and HBEGF contributed to breast cancer brain metastasis^5^. However, the comprehensive mechanisms of this process remain unclear.

Glycosylation is one of the most important post-translational modifications (PTM) and has been successfully utilized as a biomarker of many cancers^19–26^. Recently, the sialylation of Lewis X epitopes was detected and glycan levels were found to be higher in patients with high circulating tumor cells (CTCs)^27^. In addition, CD44, HPA, and Galectin were found likely to be involved in breast cancer metastasis^28–31^. However, few current studies are focusing on breast cancer brain metastasis. The Massague group also found that the ST6GALNAC5 glycogene contributes to breast cancer brain metastasis by increasing the adhesion of breast cancer cells to the blood-brain barrier^5^. To our best knowledge, this is the only glycogene which is directly confirmed to be associated with breast cancer brain metastasis.

Herein we have integrated transcriptomics, proteomics, and glycomics among different breast cancer cell lines. Correlations between these different “-omics” were studied to achieve a deeper insight of the biological molecular mechanism of breast cancer brain metastasis. In addition, we focused on the glycosylation and correlations between glycogene expression and N-glycan patterns to further investigate the relationship between glycosylation and breast cancer brain metastasis and potential glyco-biomarkers.

## Results and Discussion

### Cell line clinicopathological features

Five breast cancer cell lines (MDA-MB-231, MDA-MB-231BR, MDA-MB-361, HTB131, and HTB22) and one brain cancer cell line (CRL-1620) were employed in this study. Each cell line has a different clinicopathological feature, including phenotypes, metastatic properties, and sites (Table 1). Among them, MDA-MB-231BR (231BR) has a 100% trend to metastasize to the brain, and it cannot migrate to any other organs; the brain is not the first target for other cell lines, which can migrate to areas such as bone and lung^6,32^. 231BR is the subline of MDA-MB-231 (231). 231 was initially injected into a nude mouse, and then tumor cells from its brain metastatic site were isolated and injected into another mouse. After 6 cycles, 231BR was generated and had a specific capacity of brain metastasis. Therefore, 231BR, as a brain-seeking cell line, was special in this study and used as the reference cell line in comparison with five other cell lines. Due to its specific capacity of brain metastasis, any unique expressions in 231BR when compared to other cell lines were expected. These differences probably contributed to the penetration of cells through the blood-brain barrier, thus facilitating breast cancer brain metastasis.

**Table 1:** Immunophenotypes of six cancer cell lines.

Cell Line Name	subtype	ER	PR	HER2	tumor source	Pathology
MDA-MB-231	Basal	−	−	−	Metastasis; pleural effusion	Breast cancer
MDA-MB-231BR	Basal	−	−	−	Metastasis; brain	Breast cancer
MDA-MB-361	Luminal B	+	+	+	Metastasis; brain	Breast cancer
HTB131	Basal	−	−	−	Metastasis; pericardial effusion	Breast cancer
HTB22	Luminal A	+	+	−	Metastasis; pleural effusion	Breast cancer
CRL-1620					Brain	Brain cancer

### NGS transcriptome mapping and differential expression analysis

Total RNAs from six cell lines were applied in this study. Using the next generation sequencing (NGS) technique, overall 1.25 billion paired end reads were generated with 66X sequencing depth by sequencing the pooled cDNA libraries from the six cell lines. Function annotation against a reference human genome (Homo sapiens-build 36.1.hg18) revealed 24763 transcripts from the cell lines.

**Figure 1:**
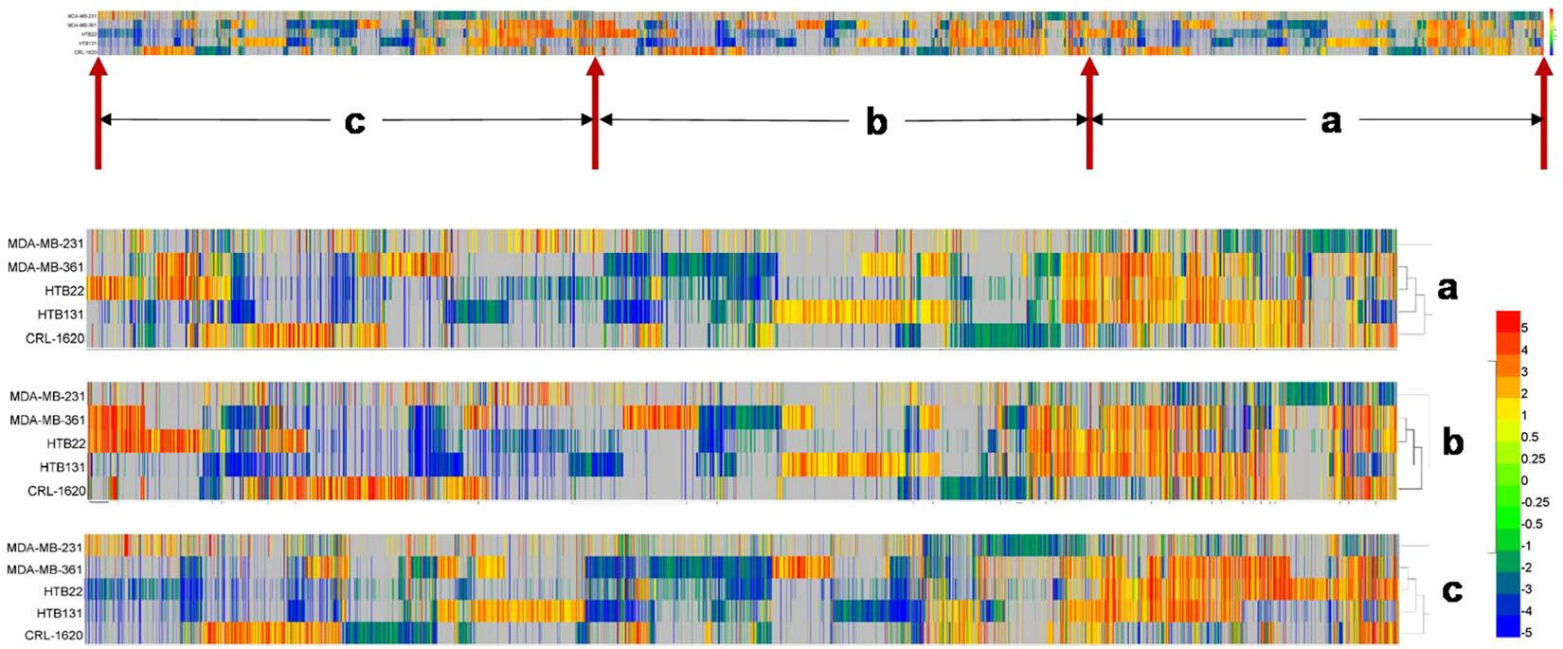
Gene differential expression patterns of five cell lines relative to MDA-MB-231BR. Distinct gene expression patterns of five cell lines were observed when compared to 231BR. (**a**–**c**) Denote the corresponding zoomed in areas on original heatmap. The color codes represent gene expression fold changes (RPKM) of each cell line relative to 231BR. Red color denotes the up-regulation of genes in other cell lines when compared to 231BR, and down-regulation in 231BR. Blue color denotes the down-regulation in other cell lines, and the up-regulation in 231BR. Gray color denotes the gene expressions had no significant difference when compared to 231BR.

Differential expression analysis was achieved by the use of QSeq software. 14551 genes were found differentially expressed in all six cell lines when they were compared with 231BR, with a fold change of more than 2 and a p-value less than 0.01. Figure 1 depicts the quantitative transcriptomic visualization of differentially expressed genes. The color codes (color bar on the right) denotes the up-regulation or down-regulation of the genes in five cell lines realtive to 231BR. Each comparison has a different expression pattern distinct from the others which denotes that there are differences among all cell lines at the transcriptomic level. 231 shows the fewest difference with 231BR (least red or blue color denotes least up or downregulated genes). Interestingly, as a brain cancer cell line, gene expression difference between CRL and 231BR were less than those between other breast cancer cell lines and 231BR. It may because both CRL and 231BR are from brain tumor sites. In addition, the number of differentially expressed genes varied among the different cell lines. There were 4922 (19.9%), 8022 (32.4%), 6585 (26.6%), 7348 (29.7%), and 8038 (32.5%) genes that demonstrated up/down-regulation in 231, 361, CRL, HTB22, and HTB131 comparisons, respectively. Compared to 231BR, 231 showed less difference than other cell lines, which was expected because 231BR is a subline of 231.

### Gene ontology (GO) analysis

The differentially expressed genes of the six cell lines were enriched by GO analysis. Fig. S1 depicts the top five biofunction enrichments among five comparisons (231BR was utilized as the baseline). From the inner circle to the outer circle are 231, 361, HTB22, HTB131, and CRL, respectively, compared with 231BR. The most significant function in all comparisons was cellular movement, suggesting that 231BR had the most significant modifications associated with cell movement. These gene expression changes of cell movement may enhance the brain metastatic ability of 231BR. In addition, CRL showed the largest difference due to its different cancer type.

### Pathway analysis

**Figure 2:**
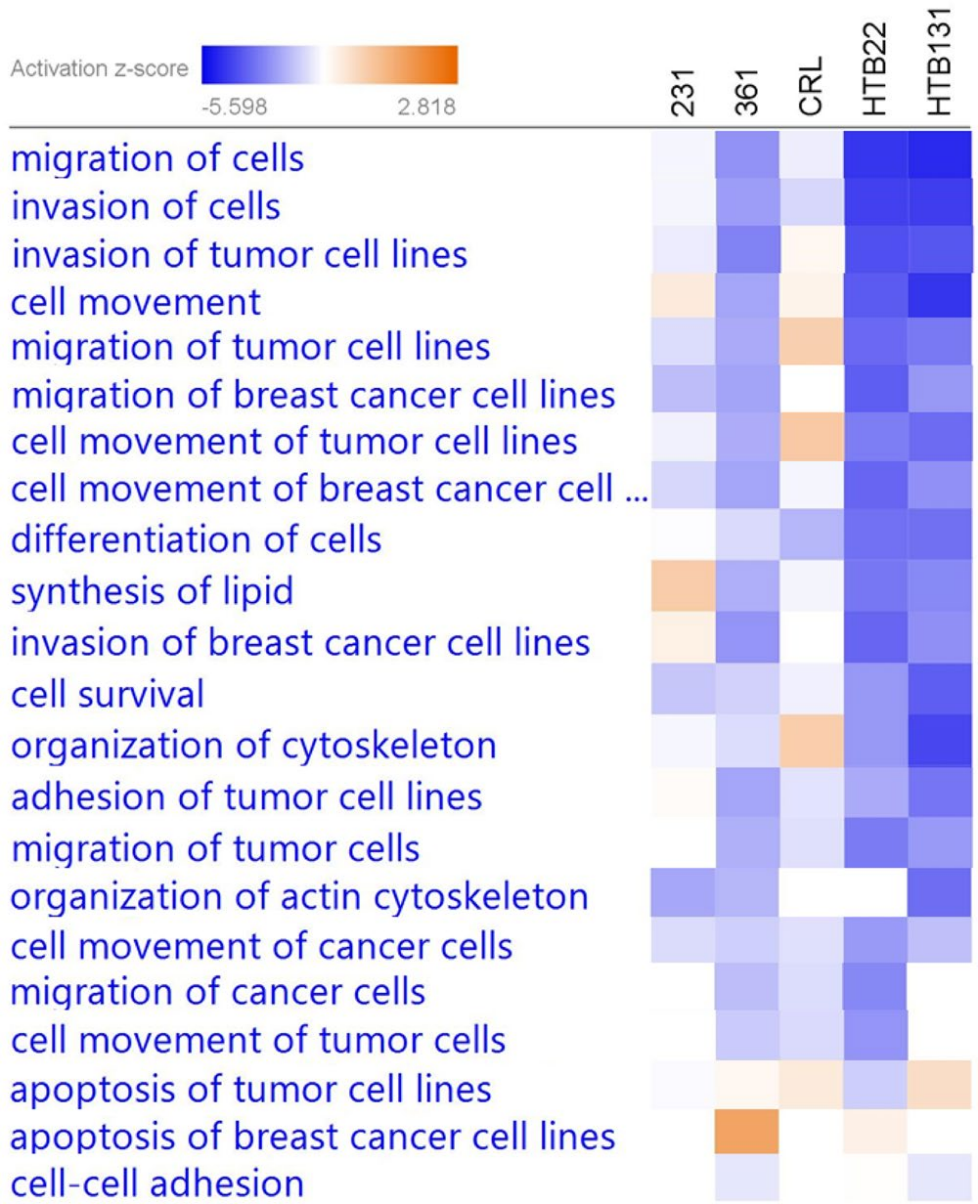
Activation/inhibition of molecular functions of different cell lines when compared to 231BR. Activation z-scores were generated by IPA based on the differential gene expressions in each cell line relative to 231BR. The [networks, functional analyses, etc.] were generated through the use of IPA (QIAGEN Inc., https://www.qiagenbioinformatics.com/products/ingenuity-pathway-analysis). Red color denotes the activation of corresponding functions in each cell line, and the inhibition in 231BR. Blue color denotes the inhibition in each cell line, and the activation in 231BR.

Pathway analysis of differentially expressed transcriptome was performed by Ingenuity Pathway Analysis (IPA, QIAGEN Inc., https://www.qiagenbioinformatics.com/products/ingenuitypathway-analysis). Again, 231BR was utilized as the baseline. The transcriptomic data from other cell lines were compared with 231BR and then uploaded to IPA. IPA searched its database and provided the pathway and biofunction enrichment, then predicted the upstream regulator or the influence on the downstream bioprocesses based on a z-score and p-value. The p-value was calculated by using Fisher’s Exact Test to estimate the significance of the clustering, and the z-score was applied to infer the activation states of predicted transcriptional regulators (upstream) and functions/pathways (downstream). Fig. S2 depicts the diseases and disorders activation status of the five cell lines compared with 231BR. The invasion of tumor, growth of tumor, and metastasis abilities of all cell lines were predicted to be inhibited when compared to 231BR, indicating that 231BR had a higher invasive and metastatic capacity over the other cell lines. Figure 2 depicts the functional activation status of the cell lines when compared with 231BR. The darker blue color denotes that the corresponding function is less active compared to 231BR. The migration of cells was predicted to be more active in 231BR when compared with other breast cancer cell lines (blue color denotes the inhibition in other cell lines, and activation in 231BR). These results suggest that the differentially expressed genes that were associated with cell migration may contribute to the high brain metastatic ability of 231BR.

**Figure 3:**
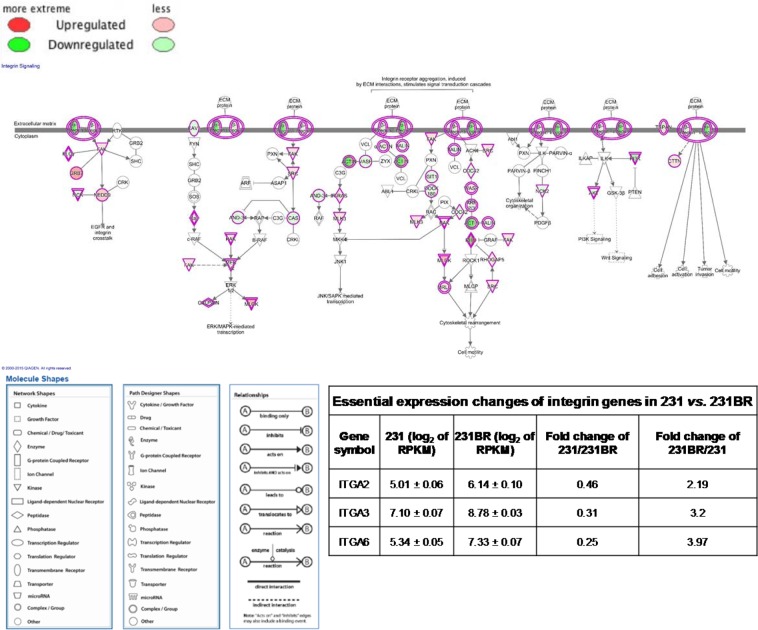
Integrin signaling pathway of 231 vs. 231BR. Green color denotes the down-regulation of genes in 231, and up-regulation in 231BR. The down-regulation of integrin in 231 (up-regulation in 231BR) may affect the cell adhesion, activation, invasion and mobility functions. The inserted table depicts the expression changes of essential integrin genes in 231 *vs*. 231BR.

231BR was derived from 231 and showed the highest similarity with 231. The expression changes between 231BR and 231 were considered to be of highest importance and probably play a role in brain metastasis. Thus, the pathway and upstream regulator analyses were focused on 231 and 231BR in this study. The integrin signaling pathway (Fig. 3) and VEGF signaling pathway (Fig. S3) were shown to be inhibited in 231 by IPA, and thus were enhanced in 231BR (green color denotes the down-regulation of gene in 231, and up-regulation in 231BR). The integrin family, as a transmembrane receptor, is one of the key elements in integrin signaling pathway that regulates many bioprocesses, such as cell cycles and cytoskeleton organization^33^. It is made of α-integrin and β-integrin that mediate cell proliferation, migration, metastasis, and survival. It has recently become clear that ligated or unligated integrin can positively or negatively influence tumor cell survival, which are often contradicted in some cases, thus facilitating tumor growth and metastasis^34^. Figure 3 depicts the integrin signaling pathway in the 231 *vs*. 231BR comparison. The up-regulation of α-integrin (including ITGA2 and ITGA3) was observed in 231BR (green color denotes the down-regulation of integrin in 231 and up-regulation in 231BR). According to IPA, the integrin signaling pathway was inhibited in 231 when compared to 231BR while it was activated in 231BR. The higher activity of the integrin signaling pathway could positively affect cell adhesion and mobility, which are important in brain metastasis. Therefore, the up-regulation of integrin may facilitate the brain metastatic ability of 231BR. As shown in Fig. S3, the VEGF signaling pathway was also predicted to be inhibited in 231, which meant more activity in 231BR. VEGF can regulate vascular permeability by activating VEGF receptors^35^. Therefore, the activation of the VEGF pathway may help 231BR cells to pass through the blood-brain barrier.

**Figure 4:**
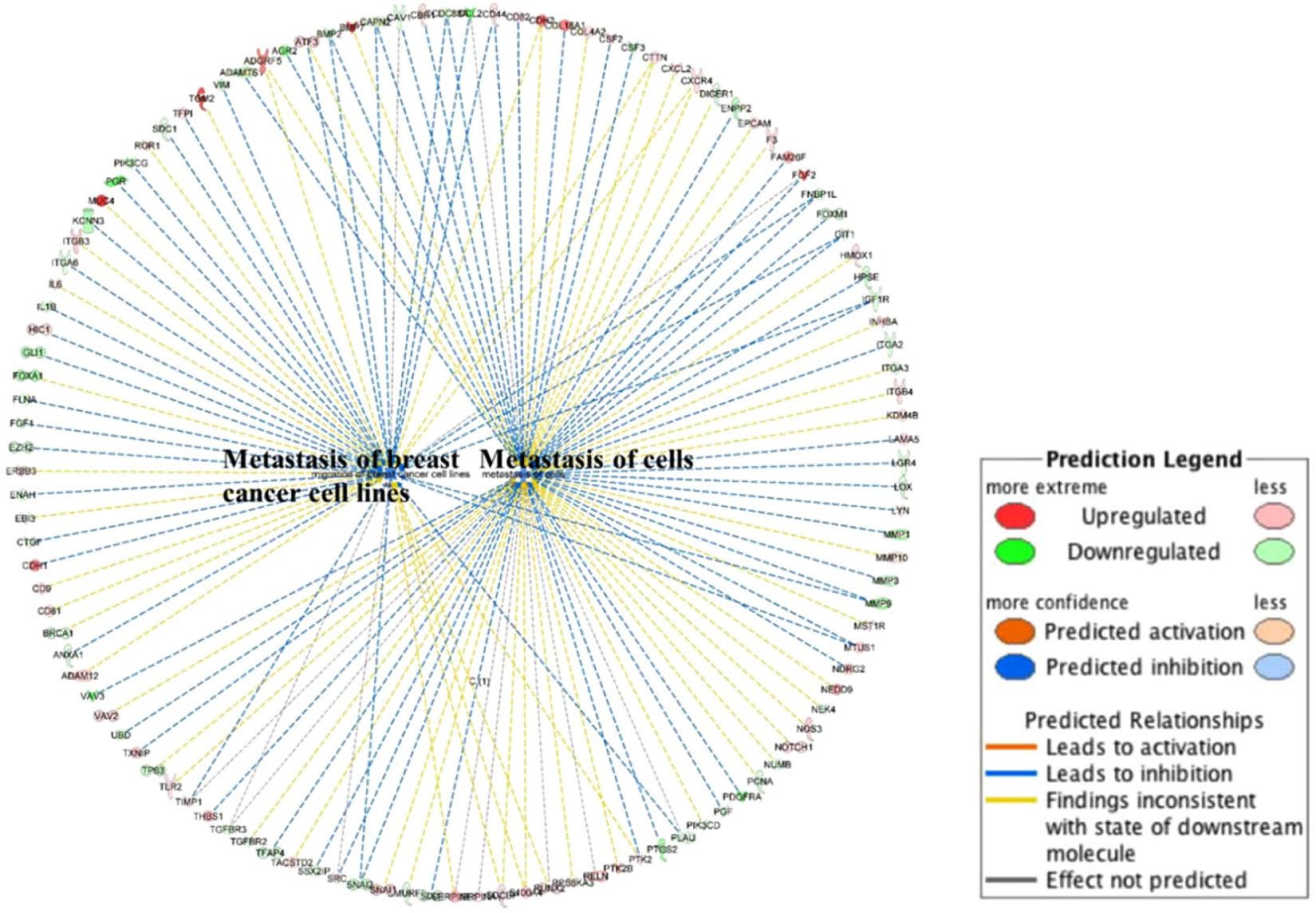
Functional annotation. Genes that exhibited different expression changes in 231 *vs*. 231BR were annotated to metastasis of breast cancer cell lines function and metastasis of cells function, respectively, using IPA. The networks and functional analyses were generated through the use of IPA (QIAGEN Inc., https://www.qiagenbioinformatics.com/products/ingenuity-pathway-analysis). Both functions were inhibited in 231 when compared to 231BR. Lines between genes and functions represent how these changes of genes affect the corresponding functions. Blue line denotes that the corresponding gene change cause an inhibition to the function in 231, and the activation in 231BR. Red line denotes that the corresponding gene change cause an activation to the function in 231, and the inhibition in 231BR, while yellow line denotes the inconsistent changes of corresponding genes and their downstream molecules (this prediction was performed by matching the gene changes observed in this experiment to those in IPA’s database).

In functional annotation, genes that are associated with specific biological functions can be grouped and the activation status of their relative functions can be predicted by the up/down-regulation of these genes. Figure 4 depicts the metastasis function annotation in 231 *vs*. 231BR. Most of the relative genes were down-regulated in 231, resulting in the inhibition of the two metastasis functions. Therefore, the metastasis of breast cancer cells and metastasis of cells were predicted to be more active in 231BR (these two functions were inhibited in 231 and activated in 231BR), meaning that 231BR had a better metastasis capacity than 231. Moreover, Fig. S4 depicts the most significant upstream regulator as MTF1, which was predicted by IPA based on the changes of observed genes. The down-regulation of MTF1 could result in the down-regulation of a series of genes, thus decreasing the activity of cell movement.

### Glycosylation gene enrichment

Aberrant glycosylation has been proven to be associated with breast cancer metastasis^7^. However, only one glycosylation gene has been demonstrated to be associated with breast cancer brain metastasis^5^. In this study, we manually selected the glycosylation genes from differentially expressed genes and investigated their expression change to find the possible glycogenes which may positively facilitate breast cancer brain metastasis. Overall, 91 glycosylation genes were selected for further study. Fig. S5 depicts the differential expressions of these glycogenes. Among them, 12 genes (ST6GALNAC2, ST6GALNAC5, FUT5, POFUT1, B4GALNT3, B4GALNT4, B4GALT6, MGAT3, MGAT4A, XYLT2, GALNT13, and PIGK) showed the same up- or down-regulation in at least four comparisons, making the expression of these genes unique in 231BR. These 12 genes can influence sialylation (ST6GALNAC2, ST6GALNAC5), fucosylation (FUT5), glycan anchor (PIGK), and complex glycan structure synthesis (B4GALNT3, B4GALNT4, B4GALT6, MGAT3 and MGAT4A), which make the unique glycan expression pattern of 231BR possible. Thus, the alteration of the expression of these genes may play important roles in breast cancer brain metastasis. However, the function of these genes need to be verified in future studies. Figure S6A depicts the functional annotation of the glycosylation genes in IPA. Most of the glycosylation genes cannot be predicted by IPA due to insufficient studies of these genes. However, based on the known correlation between glycosylation genes and their functions, sialylation and glycosylation were enhanced in 231BR and inhibited in 231, which was consistent with the cell line glycomic results (see section “*Cell Line Glycomics Analysis*”). Figure S6B shows the interactions of glycosylation genes/proteins and other related genes/proteins. The glycosylation alteration caused by the expression change of glycogenes may influence the integrin pathway and the cancer mechanism pathway *via* ITGAs.

### Cell line proteomics analysis

**Figure 5:**
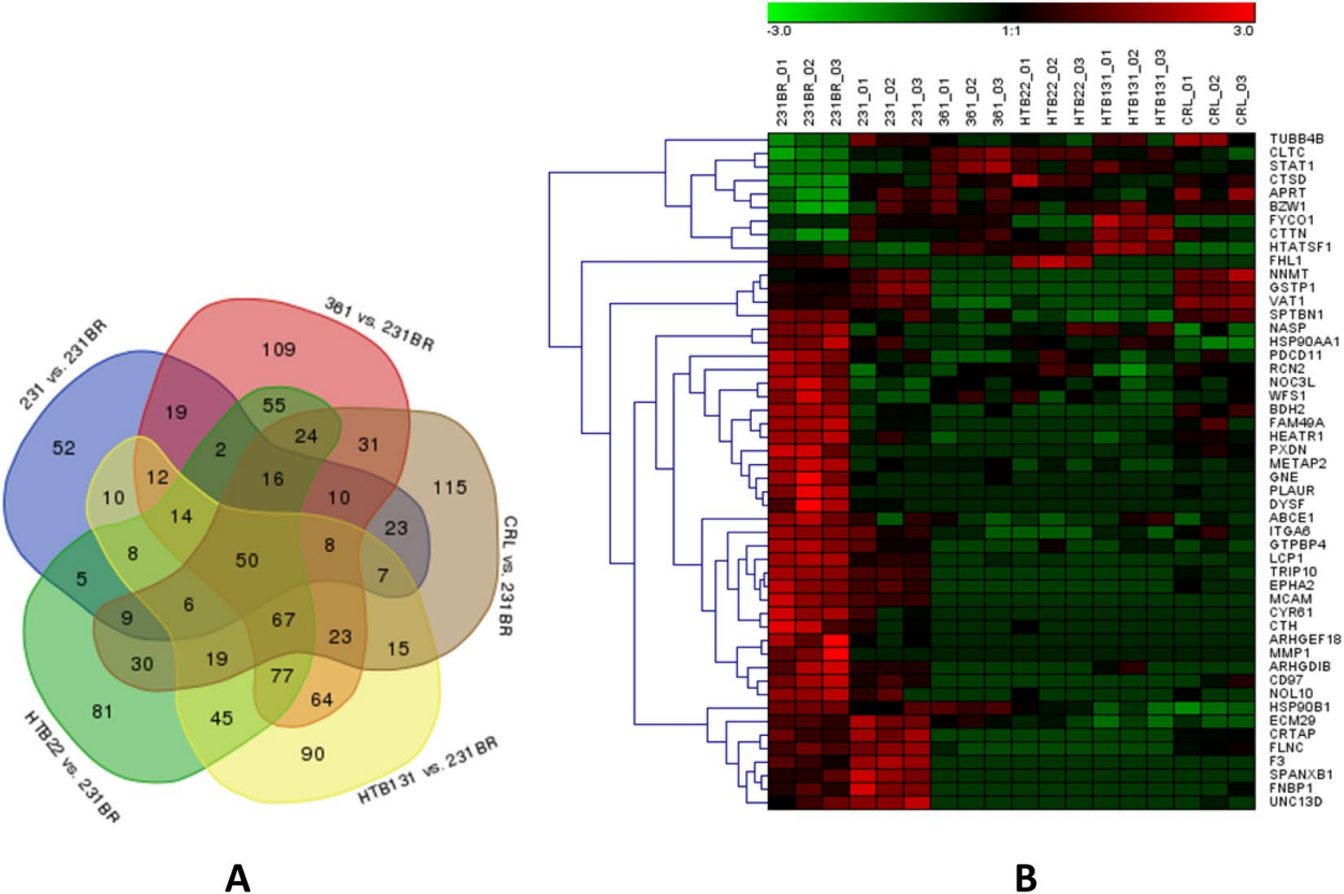
Proteins that had the significant expression alterations in five cell lines when compared to 231BR (N = 3 biological triplicates). (**A**) Venn diagram of differentially expressed proteins in cell lines compared with 231BR, with the p-value less than 0.05; (**B**) Heatmap of 50 common proteins that exhibited significant expression changes in all five comparisons. Color codes represent the expression levels of proteins across six cell lines. Red color denotes the high expression level while green color denotes the low expression level.

Unlabeled bottom-up proteomics of six cell lines were achieved using nano-C18-LC-MS/MS with a 2-hour LC gradient and 60 K MS resolution. The optimized LC condition and high MS resolution permitted a total of 1569 proteins to be identified and quantified across the six cell lines (Supplementary Information Table S1A). Brain-seeking cell line 231BR was applied as a baseline and compared with the other cell lines. In total, 1096 proteins exhibited significantly differential expressions (p < 0.05) when compared with 231BR (Supplementary Information Table S1B). Among the different cell lines, 231/231BR had the fewest differentially expressed proteins (151 proteins) due to the high similarity between 231 and 231BR. There were 581, 508, 515, and 454 significantly altered proteins (p < 0.05) in 361, HTB22, HTB131, and CRL, respectively. Figure 5A depicts the Venn diagram of the significant proteins in five comparisons. 50 proteins exhibited significant up/down-regulation in all comparisons, which made their expressions unique in 231BR (Fig. 5B). Among these, 37 proteins showed the same up- or down-regulation in all five comparisons; 30 proteins were up-regulated in 231BR, while 7 of them were down-regulated. Lymphocyte cytosolic protein 1 (LCP1) was the most significant up-regulated protein in 231BR, while clathrin heavy chain (CLTC) was the most significant down-regulated protein (Fig. S7). LCP1 belongs to the class of actin-bundling proteins. It is usually ectopically expressed in cancer cells^36^ and has been reported to promote tumor metastasis with phosphorylation^37^. In this study, we observed the obvious up-regulation of LCP1 in the brain-seeking cell line 231BR, suggesting that the up-regulation of LCP1 may play a role in breast cancer brain metastasis. CLTC is a clathrin heavy chain that participates in endocytosis, membrane trafficking, and tumorigenesis^38,39^. However, few reports have associated CLTC with cancer metastasis. Although we are unable to conclude that CLTC contributes to breast cancer brain metastasis, a significant down-regulation in brain specific cell line 231BR was observed, which may provide useful information for further studies. In addition, the up-regulation of adhesion G protein-coupled receptor E5 (CD97) was also observed in 231BR.

### Unsupervised principal component analysis (PCA)

Principal component analysis is a statistical approach to simplify high dimensional complex data sets. It uses the orthogonal transformation to convert possibly correlated variables into principal components which are linearly uncorrelated, thus displaying the similarities and differences of data groups by plots on a map^40^. Overall, 1569 proteins, including 1096 differentially expressed proteins, were submitted to PCA. Figure S8 depicts the unsupervised PCA plots of quantitative proteomic data from six cell lines. Each cell line had three biological replicates and was well-grouped. The differences between the six cell lines can be observed through primary principal component (PC1) and secondary principal component (PC2). The three biological replicates of each cell line were closely located, suggesting the satisfactory reproducibility of the cell culturing and sample preparation procedures used in this study. All cell line groups were separated, denoting the proteome differences among the different cell lines. As expected, 231BR showed the highest similarity with 231. Meanwhile, 231 and 231BR, considered to be the most invasive breast cancer cell lines, showed significant distinction from other cell lines. The brain cancer cell line CRL exhibited the largest difference from other cell lines due to its different cancer type. The PCA result suggests that the proteomic data from all cell lines are reliable and obviously distinct, which may be crucial for a better understanding of breast cancer brain metastasis.

### Correlation between proteome and transcriptome

A biosystem is a complicated and self-adjustable system. After transcription, mRNAs experience translation and post-translational modification to generate functional and structural proteins and enzymes. Many regulatory processes occur between mRNAs and proteins. Thus, identifying the correlation between transcriptome and proteome is necessary to achieve a better understanding of bioprocesses, herein emphasizing brain metastasis. Proteins which had significantly differential expression (p < 0.01) were selected to correlate with their transcripts. Most proteins exhibited the same regulation trend as their transcription changes (Supplementary Information Table S2). On average, 83.8% of protein expressions matched the gene expressions. 79.3%, 85.4%, 87.1%, 84.2%, and 83.2% of the differential expressed proteins showed the same expression changes as their transcripts in 231, 361, HTB131, HTB22, and CRL compared with 231BR, respectively. Although the fold change of protein expression and gene expression had a slight difference, it could be expected due to the complicated regulations between genes and proteins and the existence of isoenzymes. Figure S9 depicts the correlation between significant protein expression changes (p < 0.01) and transcript expression changes in 231 *vs*. 231BR. 73 out of 92 proteins showed the same up- or down-regulation as their transcripts (Fig. S9). 40 proteins were observed to be up-regulated in 231BR, while 52 were down-regulated. Among the up-regulated proteins, 2′,3′-cyclic nucleotide 3′ phosphodiesterase (CNP) exhibited the largest expression fold change, which was 14 times increased, although the increase of its gene expression was not so large. CNP was reported as overexpressed in the brain when compared with other tissues^41^. It was initially reported as phosphodiesterase, but has recently been reported to have an association between its differential expression and glioblastoma patient survival^42^. The increase of both CNP transcript and protein in 231BR can most likely be associated with its specific brain metastatic capacity. Interestingly, when looking into the correlation between protein expressions and corresponding gene expressions in all cell lines realtive to 231BR, pearson correlation coefficients between protein and gene expression changes were less than 0.77, as shown in Fig. S10. Silimlar to 231/231BR comparison, most of proteins and genes exhibited the same up- or down- regulation trends. The low pearson correlation coefficient maybe due to the fact that the fold changes of genes did not match the fold changes of proteins (e.g. the fold change of CNP protein was 14.2 times, while the fold change of CNP gene was only 1.2 times).

According to GO clustering analysis, differential expression proteins were enriched into different molecular and cellular functions with a high significance (p < 1E-07). Similar to their differential expressed genes (Fig. S1), proteins associated with cell movement, cell growth and proliferation, cell death and survival, and cellular assembly and organization exhibited differential expression compared to 231BR (Fig. S11). The GO results stressed the importance of genes/proteins associated with cell movement in the breast cancer brain metastasis process. When we examined proteins with a significance p-value of less than 0.05, we also observed the up-regulation of α-integrin (ITGA) in 231BR, which affected the cell movement and cell adhesion *via* many pathways based on IPA, such as the integrin signaling pathway (Fig. S12, green color denotes the down-regulation of integrin in 231, and up-regulation in 231BR). When we considered cell-to-cell signaling and interactions which were generated by IPA (Fig. S13), the integrin proteins also exhibited a central role in the network. This suggests that they might be important in relative bioprocesses, including brain metastasis.

Although many important correlations between transcriptome and proteome from six cell lines were observed, we could not detect glycosylation proteins in cell line proteomics. The reason may be because the expression level of glycosylation proteins (enzymes) are relatively low in cells, such that it is difficult to detect them using our LC-MS/MS conditions without specific enrichment. Therefore, we investigated the N-glycomic alterations among different cell lines to achieve a better understanding of the functions of glycans and glycogenes in breast cancer brain metastasis.

### Cell line glycomics analysis

N-Glycomic Analysis of six cell lines was performed using LC-MS/MS. The most obvious advantages of the LC-ESI-MS method are its high sensitivity and adequate structural information. The sample preparation protocol provided a relatively complete release of N-glycans from cells; the solid-phase permethylation enabled higher ionization efficiency of neutral and sialylated glycans and better separation on reversed phase LC with a minimum sample loss; the on line purification removed salts and impurities, which provided a better ionization of glycans; the LTQ Orbitrap Velos mass spectrometer offered a high resolution and mass accuracy, and made the identification of low abundant glycan structures possible. All of the elements of this LC-ESI-MS method contributed to the sensitive and reproducible detection of N-glycans released from cell lines. Overall, 63 structures were identified from cell line samples, with a mass accuracy less than 5 ppm. The number of identified N-glycans was obviously higher than other techniques such as MALDI-TOF^43^. Among these structures, 60 N-glycans exhibited significantly different expressions in at least one comparison (p < 0.05) (Supplementary Information Table S3). The highest number of N-glycans were identified in 231BR (63 N-glycans), followed by CRL and 231 (61 and 56 N-glycans, respectively). However, there were fewer N-glycans detected in 361, HTB131, and HTB22 (48, 25, and 33 N-glycans, respectively). The superior glycan numbers in 231BR and 231 are likely because they are considered to be the most invasive breast cancer cell lines^44^. This result supports the contribution of glycosylation to breast cancer brain metastasis.

### Correlation between transcriptome and glycome

**Figure 6:**
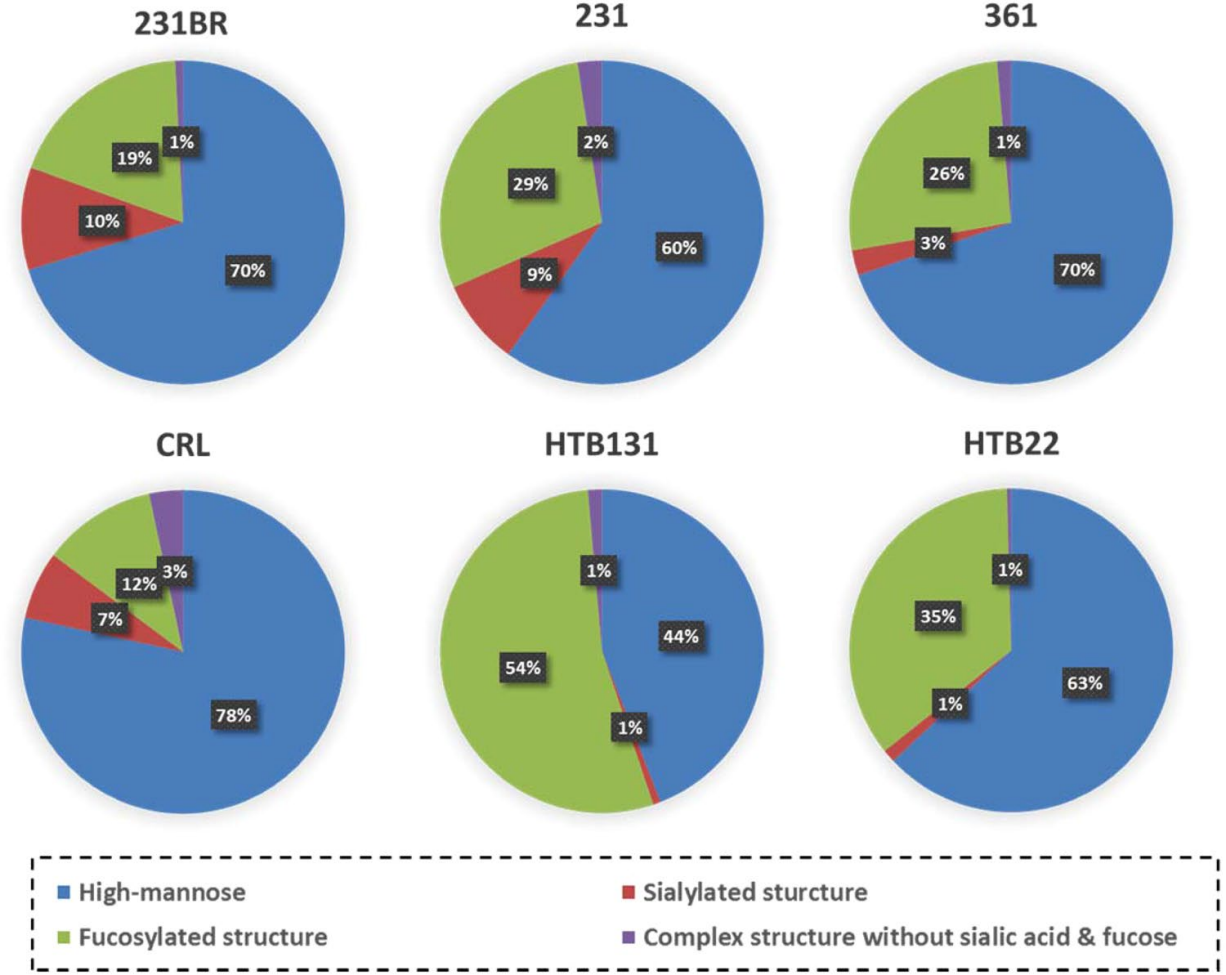
Distribution changes of different glycan types from six cell lines.

Because of the unique brain metastatic capacity of 231BR, it was used as the baseline to compare with the other cell lines. The significant glycosylation alterations in 231BR may contribute to its brain metastasis. The overall intensity of N-glycans identified in 231BR was higher than in other cell lines (Supplementary Information Table S3), which suggests a higher glycosylation level in brain metastasis. Detailed information can be determined when a closer investigation is conducted between these cell lines, especially when identified N-glycans are grouped by their composition characteristics. Figure 6 shows the distribution of different glycan types in different cell lines. 231BR and 231 have more sialylated structures than other cell lines. According to glycogene expressions (Fig. S5), most of the genes associated with sialylation (such as ST6GALNAC5 and ST6GAL1) were down-regulated in other cell lines, which resulted in the higher sialylation of 231BR and 231. Moreover, the sialylated glycans in 231BR were statistically more than 231 (p < 0.04). Although some sialylation genes were observed to be up-regulated in 231, two important sialylation genes, ST6GALNAC5 and ST6GAL1, were 13.5 fold and 7.0 fold down-regulated, respectively; only one relative gene had a significant up-regulation (ST6GALNAC2, 14.3 fold up-regulated). Considering that ST6GALNAC5 has been demonstrated to contribute to the penetration of breast cancer cells through the blood-brain barrier, thus facilitating the brain metastasis^5^, ST6GALNAC5 may have a higher catalyzing activity than its isoenzyme ST6GALNAC2. In contrast, ST6GALNAC2 was reported to be a suppressor of breast cancer metastasis^45^. The down-regulation of ST6GALNAC2 may also contribute to breast cancer brain metastasis by helping cancer cells to pass through the blood-brain barrier. The up-regulation of ST6GALNAC5 and ST6GAL1 resulted in a higher sialylation level in 231BR. In addition, sialylated glycan structures appear to increase with the progression of the cancer stage^43^ and have the capacity to mediate cellular interactions^46^. Therefore, we believe that sialylation and sialylated glycans play an important role in breast cancer brain metastasis.

**Figure 7:**
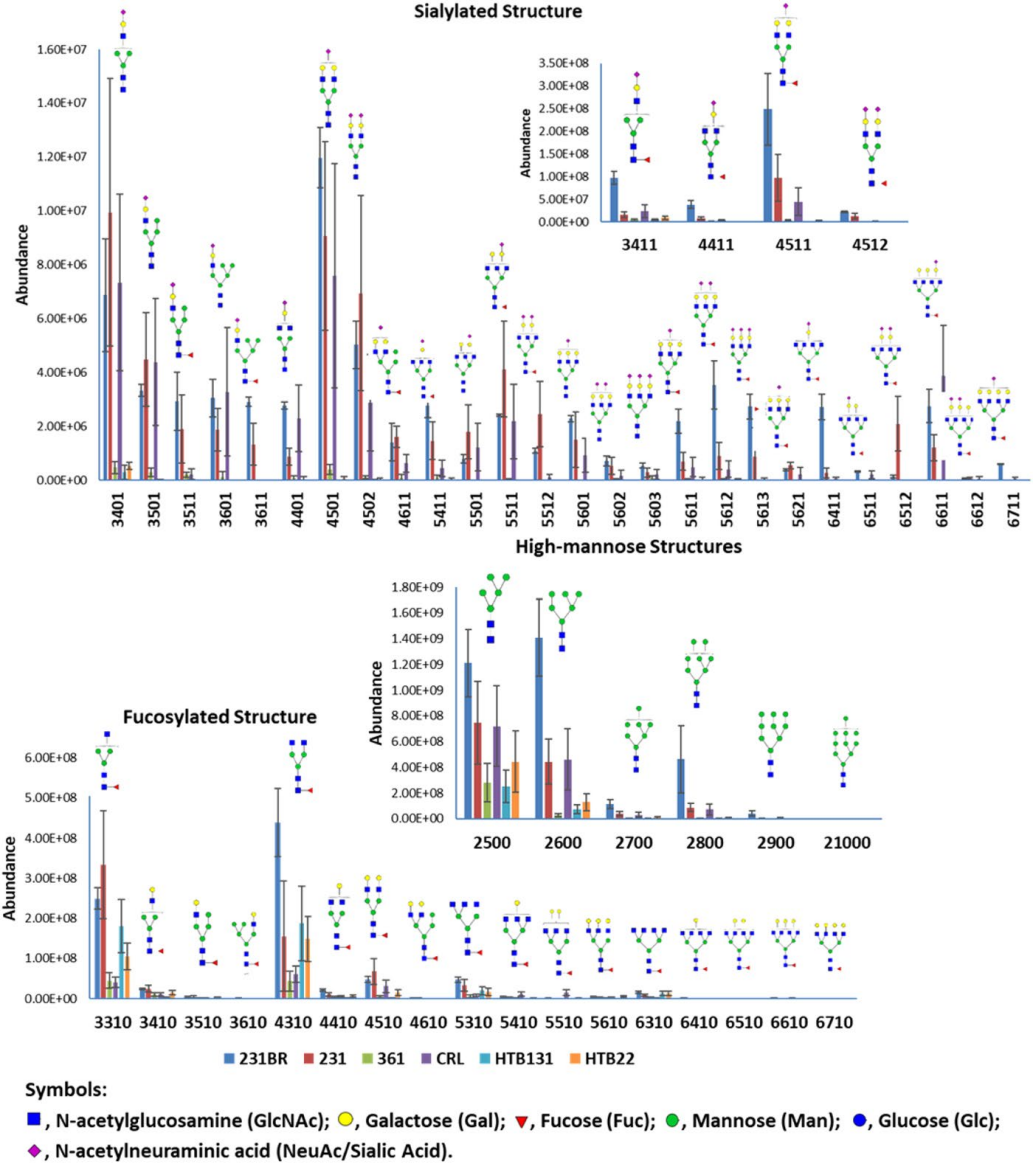
Absolute abundance and putative glycan structures from six cell lines using LC-MS/MS. Error bars denote the standard deviations from 3 biological tripilicates. Glycan composition is presented by a 4-digit code. Nomenclature: a 4-digit code is employed to represent glycan structures. The 1^st^ number denotes the number of N-acetylglucosamine (HexNAc); the 2^nd^ number denotes the number of hexose (Hex); the 3^rd^ number denotes the number of fucose (DeoxyHex); the 4^th^ number denotes the number of sialic acid (NeuAc). For example, 4512 represents glycan HexNAc_4_Hex_5_DeoxyHex_1_NeuAc_2_.

Regarding individual N-glycans, the glycan expressions (distributions) were altered among the different cancer cell lines (Fig. 7 and Supplementary Information Table S3). Most of the glycan structures showed no significant change between 231 and 231BR (p > 0.05). However, highly sialylated structures such as HexNAc_5_Hex_6_DeoxyHex_1_NeuAc_1_, HexNAc_5_Hex_6_DeoxyHex_1_NeuAc_2,_ and HexNAc_5_Hex_6_DeoxyHex_1_NeuAc_3_ were overexpressed in 231BR. For the high abundant sialylated glycans, as shown in the inset of Fig. 7, 231BR also exhibited a significantly higher expression level (p-value < 0.05) in all four glycans (HexNAc_3_Hex_4_DeoxyHex_1_NeuAc_1_, HexNAc_4_Hex_4_DeoxyHex_1_NeuAc_1_, HexNAc_4_Hex_5_DeoxyHex_1_NeuAc_1_, and HexNAc_4_Hex_5_DeoxyHex_1_NeuAc_2_). The overexpression of these glycans may be important for breast cancer brain metastasis. In addition, the sialylated structures in 231 and 231BR were predominant compared to other cell lines, which matched the distributions of different glycan types in cell lines (Fig. 6). Similar results can be observed for high-mannose structures. 231BR expressed more high-mannose structures, especially MAN6. According to transcriptomic data, most of the mannosidase genes (such as MANBA or MAN1) were up-regulated in the 361, HTB131 and HTB22 cell lines. Since mannosidase trims a high-mannose structure to form a core structure, the up-regulation of these genes may result in the decrease of high-mannose structures. For the fucosylated structures, most of the fucosylated glycans didn’t show any significant difference, especially the low abundant structures. In addition, according to Fig. 6, the distribution of the fucosylated types were relatively low in 231BR. This result may be caused by the down-regulation of the fucosylation genes (FUT) in 231BR (Fig. S5).

Through the correlation of transcriptomics, cell line glycomic analysis has revealed the essential role of sialylation and sialylated N-glycans in breast cancer brain metastasis. Seven structures, both fucosylated and sialylated (HexNAc_5_Hex_6_DeoxyHex_1_NeuAc_1_, HexNAc_5_Hex_6_DeoxyHex_1_NeuAc_2_, HexNAc_5_Hex_6_DeoxyHex_1_NeuAc_3_, HexNAc_3_Hex_4_DeoxyHex_1_NeuAc_1_, HexNAc_4_Hex_4_DeoxyHex_1_NeuAc_1_, HexNAc_4_Hex_5_DeoxyHex_1_NeuAc_1_, and HexNAc_4_Hex_5_DeoxyHex_1_NeuAc_2_) may have contributed to the brain metastatic process.

## Conclusion

In this study, we investigated and correlated transcriptomics, proteomics, and glycomics of five breast cancer cell lines and one brain cancer cell line. The brain-seeking cell line 231BR was used as a reference to compare with the other cell lines. Unique expressions in 231BR were highlighted as potential biomarkers in breast cancer brain metastasis. IPA of transcriptomic data suggested the highest levels of cell movement, invasion, and metastatic ability in 231BR. With agreement between transcriptomics and proteomics, it appears that the integrin signaling pathway with up-regulation of ITGA may play an important role in breast cancer brain metastasis. Alpha-V integrin (ITGAV) has been associated with brain metastasis, but the relationship between ITGA2/3 and breast cancer brain metastasis has not well known yet. The up-regulation of ITGA2 and ITGA3 observed in this study in brain seeking breast cancer cell line 231BR may provide a potential indicator for breast cancer metastasis, and better understand the role integrin plays in this critical bioprocess. 12 glycosylation genes that could affect glycan patterns were observed to have unique expressions in 231BR, and the protein-protein interactions of these genes showed their possible correlation to the integrin pathway. Based on glycomics results, the most glycans were identified in 231BR, denoting the significance of glycosylation in breast cancer brain metastasis. A higher sialylation level may be essential in breast cancer metastasis. Several highly sialylated glycans including HexNAc_5_Hex_6_DeoxyHex_1_NeuAc_1_, HexNAc_5_Hex_6_DeoxyHex_1_NeuAc_2_, HexNAc_5_Hex_6_DeoxyHex_1_NeuAc_3_, HexNAc_3_Hex_4_DeoxyHex_1_NeuAc_1_, HexNAc_4_Hex_4_DeoxyHex_1_NeuAc_1_, HexNAc_4_Hex_5_DeoxyHex_1_NeuAc_1_, and HexNAc_4_Hex_5_DeoxyHex_1_NeuAc_2_ were observed to be overexpressed in 231BR, suggesting an association of these glycans with breast cancer brain metastasis. The increase of sialylation level and some of the sialylated glycans such as HexNAc_5_Hex_6_DeoxyHex_1_NeuAc_2_ have been observed in breast cancer progression^47^. In addition to breast cancer progression, in this study, the increase of sialylation and aforementioned seven glycans could be considered as potential indicators for breast cancer brain metastasis. These results provide important clues for a better understanding of breast cancer brain metastasis. Although these unique expressions of genes/proteins/glycans were observed in breast cancer brain seeking cell line, we cannot conclude that they are the biomarkers of breast cancer brain metastasis due to the limited sample size. They need to be further investigated and verified using clinical samples.

## Methods

### Materials and reagents

Breast cancer cell line MDA-MB-231, MDA-MB-361, HTB131, HTB22 and CRL-1620 were purchased from American Type Culture Collection (ATCC). Breast cancer cell line MDA-MB-231BR was a generous gift from Dr. Paul Lockman (Texas Tech University Health Sciences Center, School of Pharmacy, Amarillo, TX). RPMI-1640 Medium, Eagle’s Minimum Essential Medium (EMEM), Dulbecco’s Modified Eagle’s Medium (DMEM), Fetal Bovine Serum (FBS) and Penicillin-Streptomycin Solution (100X) were purchased from ATCC. Corning Trypsin EDTA 1 × (0.25% Trypsin/2.21 mM EDTA) was purchased from Fisher. Cell culturing plates and flasks were purchased from Corning. Mammalian total RNA Extraction Kit was purchased from Sigma-Aldrich. Borane-ammonia complex, dimethyl sulfoxide (DMSO), sodium hydroxide beads, iodomethane, and formic acid (FA) were obtained from Sigma-Aldrich (St. Louis, MO). PNGase F and digestion buffers, including 10 × G7 reaction buffer (0.5 M sodium phosphate), RXN buffer, 10× glycoprotein denaturing buffer (5% SDS, 0.4 M DTT), and NP-40 were acquired from New England Biolabs (Ipswich, MA). HPLC grade methanol, isopropanol, and acetic acid were procured from Fisher Scientific (Pittsburgh, PA). Acetonitrile was obtained from JT Baker (Phillipsburg, NJ). HPLC grade water and sodium hydroxide were purchased from Mallinckrodt Chemicals (Phillipsburg, NJ).

### Cell cultivation

MDA-MB-231 and HTB131 were grown in RPMI-1640 Medium (with 10% FBS and 2% Penicillin-Streptomycin); MDA-MB-231BR and CRL-1620 were grown in DMEM (with 10% FBS and 2% Penicillin-Streptomycin); MDA-MB-361 was grown in RPMI-1640 Medium (with 20% FBS and 2% Penicillin-Streptomycin); HTB22 was grown in EMEM (with 10% FBS and 2% Penicillin-streptomycin). Cell lines were taken out from liquid nitrogen and incubated at 37 °C with 5% CO_2_ for 7 days. When cell confluence reached 80%, cells were subcultured into 175 cm^2^ flask and incubated for another 3–7 days. After reaching 80% cell confluence, cells were washed by PBS and then detached by 5 mL trypsin-EDTA solution at 37 °C for 5 min. Then 10 mL of fresh medium was added to neutralize trypsin and cells were collected by centrifugation. These collected cells were used for transcriptomic, proteomic, and glycomic analysis.

### Transcriptomics analysis

Fresh cells were washed by PBS and then total RNAs were extracted using the Mammalian total RNA Extraction Kit, following vendor’s instruction. 5 μL of total RNA solution was taken out to do a concentration and quality measurement. RNA concentration and quality were measured by a Nanodrop 1000 instrument. Total RNAs which had the OD_260_/OD_280_ within 1.9–2.2 were considered to be good quality and were utilized for the further transcriptomic analysis. Each cell line had three biological replicates.

After quantification of total RNA, a multiplexing method was employed for the high throughput RNA sequencing. All 18 different samples from different cell lines (6 cell lines with 3 biological triplicates of each cell line) were ligated with different combinations of indexes. Adaptor ligated cDNA libraries were prepared using the NeoPrep automated microfluidic instrument with TruSeq Stranded RNA Library Prep Kit (Illumina). PCR enriched libraries were quantified by Qubit and equimolar indexed libraries (different samples had different indexes for multiplexing) were pooled. Pooled libraries were qualitatively checked using the Agilent Tapestation 2200 and quantified using Qubit. The libraries were then diluted to a final concentration of 9 pM and spiked with 2% phiX libraries (Illumina control). Illumina HiSeq. 2500 was used for next generation transcriptomic sequencing (NGS). The reads generated from different samples could be distinguished based on their different indexes. The quality of the reads were determined by FastQC software. The reads were quality filtered and mapped on the human genome using NGen software (DNA STAR, Madison, WI). RPKM normalization was then performed prior to the quantification of gene expression by QSeq (DNA STAR). Genes showing >2.0 fold change and p < 0.01 were selected as differentially expressed genes and mapped into biological pathways using Ingenuity Pathway Analysis (IPA) software. Differentially expressed gene cluster annotation and function enrichment were achieved by DAVID. Glycosylation pathways were analyzed by KEGG.

### Proteomic analysis

Three biological replicates of cells from different cell lines were suspended in 50 mM ABC buffer (containing 5% SDC) and then homogenized by a beads beater homogenizer. After lysing, 5 μL of cell lysate was taken out for protein assay using the micro BCA method (Thermo Scientific/Pierce, Rockford, IL) according to the manufacturer’s instruction. The remaining cell lysate was denatured in an 80 °C water bath for 30 min. Then the denatured proteins were reduced by 5 mM DTT at 60 °C for 45 min, alkylated by 20 mM IAA at 60 °C in darkness for 45 min, followed by quenching the reaction by adding another 5 mM DTT and incubating at 60 °C for 30 min. Next, protease Trypsin-Lys C (Promega, MS gold grade) was added to the sample with a 1:25 enzyme to protein ratio and incubated at 37.5 °C for 18 h. After tryptic digestion, formic acid was added (final concentration is 0.5%) to remove the SDC^48^. After centrifugation at max speed for 10 min, supernatant which contained peptides was collected and dried. Samples were then resuspended with 5% ACN water and ready for proteomic analysis.

Proteomics analysis was performed by LC-MS/MS (Thermo Ultimate 3000 nano system coupled with LTQ Orbitrap Velos)^49^. The online purification was performed using a C18 Acclaim PepMap 100 trapping column (75 µm I.D. × 2 cm, 3 µm particle sizes, 100 Å pore sizes, Thermo Scientific, San Jose, CA). Peptide separation was achieved using a C18 Acclaim PepMap RSLC column (75 µm I.D. × 15 cm, 2 µm particle sizes, 100 Å pore sizes, Thermo Scientific, San Jose, CA). 1 μg of proteins was injected for the analysis under 60,000 resolution. A 120-minute LC method was used for the separation of the digested peptide sample. Briefly, the LC gradient of solvent B in LC-MS/MS analysis was: 5% over 10 min, 5–20% over 55 min, 20–30% over 25 min, 30–50% over 20 min, 50–80% over 1 min, 80% over 4 min, 80–5% over 1 min and 5% over 4 min. Two scan events were applied for the data-dependent acquisition. The first scan event was a full MS scan at a resolution of 60 K. The second scan event was CID MS/MS scan which fragmented the top 10 intense precursor ions that were selected from the first scan, using the normalized collision energy (CE) of 35%, Q value of 0.25, and 10 ms activition time.

Proteomic Data was then converted to a mascot file by Proteome Discover version 1.2 software (Thermo Scientific, San Jose, CA) and then searched against a UniProt database (2014_06, Homo sapiens, 20214 entries) in MASCOT version 2.4 (Matrix Science Inc., Boston, MA). The MASCOT result was verified using Scaffold (version Scaffold_3.6.3, Proteome Software Inc., Portland, OR). Peptide identification probability and protein identification probability were set to 95% and 99%, respectively, and the matched peptide number was 2. A normalized spectra count was applied for quantitation. Then an additional filter was utilized to remove the proteins which were detected only once in three replicates. Quantitative proteomic data sets from different cell lines were compared and submitted to IPA for pathway analysis and protein-protein interactions.

### Glycomic analysis

Three tubes of independently harvested breast cancer cells from each cell line were applied in this research. Each tube contains approximately 10^6^ cells. Then each tube is resuspended by a PBS buffer (with 5% SDC) to 200 μL. Next, cell samples were sonicated in iced water for 60 minutes to open the cell and expose the cytosolic proteins. After sonication, 5 uL solution was taken out for protein assay using a BSA protein assay kit (Thermo Scientific, Pittsburgh, PA), which is an essential step for normalization injection amount by protein amount. The remaining 95 μL solution was subjected to the 80 °C water bath (Thermo Scientific, Pittsburgh, PA) for 30 minutes for denaturization. The solution was cooled down to ambient temperature and 5 μL of ten-times diluted PNGase F enzyme was added to each vial. The samples were incubated in a 37 °C water bath for 18 hours. After PNGase F digestion, 2 μL of formic acid was added to the sample solution to precipitate SDC. Then supernatants were collected after centrifuging at maximum speed for 10 min and dried. Dried samples were dissolved by 50 μL of water and dialyzed overnight with the MWCO 500–1000 to remove salt. After dialysis, samples were dried and then resuspended in 90% ethanol to remove protein. After standing in −20 °C for 30 min, samples were centrifuged and supernatants (purified N-glycans) were collected and dried. The purified N-glycans were reduced as per the protocol previously reported^50^, 20 μL solution containing 10 μg/μL ammonium-borane complex was added to each vial, the vials were incubated in a 60 °C water bath for 60 minutes, and then the mixture was washed by methanol to remove excess borane salts. After that, the reduced N-glycans were permethylated by utilizing solid-phase permethylation^51–54^. Sodium Hydroxide beads were added to spin columns (Harvard Apparatus, Holliston, MA), dried samples were resuspended in 30 μL Dimethyl sulfoxide (DMSO) and 1.2 uL water mixed with 20 μL Iodomethane, and the mixture was added to the spin column from the top. After 25 minutes’ incubation, another 20 μL Iodomethane was added to the column and incubated for another 15 minutes. The spin columns were centrifuged (Thermo Scientific, Pittsburgh, PA, USA) at 1.8k rpm for 2 minute, and then 30 μL ACN was added to the columns for the final elution. These elutes were collected and dried under vacuum (Labconco, Kansas City, MO). Finally, the samples were resuspended by 20% ACN water solution (with 0.1% formic acid) to 24–48 μL, corresponding to their protein amount as determined by BSA protein assay.

A Dionex Ultimate 3000 nano LC system (Thermo Scientific, Pittsburgh, PA, USA) was utilized in the LC-MS analysis. The separation was accomplished on an Acclaim C18 nano column (Thermo Scientific, Pittsburgh, PA, USA) under a flow rate of 350 μL/min. An optimized gradient elution condition was applied. The LTQ Orbitrap Velos (Thermo Scientific, Pittsburgh, PA, USA) was utilized for the detection of N-glycans, and the mass spectrometry was operated at positive mode with m/z range of 700–2,000. The instrument was set at data dependent acquisition mode with 8 MS/MS scans.

The analyte peaks were extracted from the full MS scans by using Xcalibur Qual Browser (Thermo Fisher Scientific) with a 10 ppm mass tolerance. The identification and quantitation of N-glycans were performed by an in-house software, Multiglycan, with a 5 ppm mass tolerance and manually checked based on the full MS and MS2.

### Integrated “Omics” analysis

Transcriptomic, proteomic, and glycomic data were correlated to each other to find the inner relationship among those different level changings and the possible biological mechanisms of breast cancer brain metastasis.

## Supplementary information

Supplementary Information

Supplementary Table S1

Supplementary Table S2

Supplementary Table S3

## Data Availability

Transcriptomics data were submitted to NCBI bioproject PRJNA383270; BioSample accessions: SAMN06806180, SAMN06806181, SAMN06806182, SAMN06806183, SAMN06806184, SAMN06806185. Proteomics data are available *via* ProteomeXchange with identifier PXD010506.
