# Supplementary material for: Integrated Transcriptomics, Proteomics, and Glycomics Reveals the Association between Up-regulation of Sialylated N-glycans/Integrin and Breast Cancer Brain Metastasis
Source: Sci Rep. 2019 Nov 22;9:17361. doi: 10.1038/s41598-019-53984-8 (PMC6874669; doi:10.1038/s41598-019-53984-8)
Supplement: Supplementary file 1 — Supplementary Information [file 41598_2019_53984_MOESM1_ESM.docx]

**Supplementary Information**

**Integrated Transcriptomics, Proteomics, and Glycomics Reveals the Association between Up-regulation of Sialylated N-glycans/Integrin and Breast Cancer Brain Metastasis**

Wenjing Peng^1^, Rui zhu^1^, Shiyue Zhou^1^, Parvin Mirzaei^1^, Yehia Mechref^1^*

^1^ Department of Chemistry and Biochemistry, Texas Tech University, Lubbock, TX

***Corresponding Author**

Department of Chemistry and Biochemistry

Texas Tech University

Lubbock, TX 79409-1061

Email: yehia.mechref@ttu.edu

Tel: 806-742-3059

Fax: 806-742-1289

**Table S1**. **A**. Quantitation of proteins from six cell lines; **B**. Quantitation of proteins which had significantly differential expressions.

**Table S2**. Correlation of significantly differentially expressed proteins with their gene expressions.

**Table S3**. Absolute abundance and putative glycan structures of six cell lines.

**Figure Legend**

**Figure S1.** Diseases and Biofunctions enrichment of five cell lines compared with 231BR. From inner circle to outer circle are 231, 361, HTB22, HTB131, CRL, respectively, compared with 231BR. The most significant function in all cell lines are cellular movement. The other four functions in top 5 varies among different cell lines. CRL has the biggest difference.

**Figure S2.** Comparison of diseases and disorders in IPA among cell lines. Data were analyzed through the use of IPA (QIAGEN Inc., <https://www.qiagenbioinformatics.com/products/ingenuitypathway-analysis>). Red color denotes the inhibition of relative functions in each cell line when compared to 231BR and activation in 231BR, and blue color denotes the inhibition in each cell lines and the activation of relative functions in 231BR.

**Figure S3.** VEGF signaling pathway. Data were analyzed through the use of IPA (QIAGEN Inc., <https://www.qiagenbioinformatics.com/products/ingenuitypathway-analysis>). Green color denotes the down-regulation in 231 compared to 231BR and the up-regulation in 231BR. Red color denotes the up-regulation in 231 and the down-regulation in 231BR. The inserted table depicts the essential expression changes of VEGF genes (N = 3 biological triplicates).

**Figure S4.** Regulator effect analysis in 231 *vs.* 231BR. Green color denotes the down-regulation of genes in 231 and up-regulation in 231BR. Blue color denotes the inhibition of the gene expressions or functions in 231 and activation in 231BR.

**Figure S5.** Glycosylation gene differential expression pattern. Color coding represents the fold changes of genes expressions (RPKM) in other cell lines compared to 231BR. Data were analyzed through the use of IPA (QIAGEN Inc., <https://www.qiagenbioinformatics.com/products/ingenuitypathway-analysis>). Green color denotes the up-regulation in other cell lines (down-regulation in 231BR). Red color denotes the down-regulation in other cell lines (up-regulation in 231BR).

**Figure S6.** IPA of glycosylation genes. Data were analyzed through the use of IPA (QIAGEN Inc., <https://www.qiagenbioinformatics.com/products/ingenuitypathway-analysis>). **A**. functional annotation of glycosylation genes; **B**. glycosylation gene network. Green color denotes the down-regulation of genes in 231 when compared to 231BR and the up-regulation in 231BR. Blue color denotes that the inhibition of the function in 231 and the activation in 231BR. Blue line denotes that the corresponding gene change cause an inhibition to the function. Black line denotes that the effect of gene change cannot be predicted to the function by IPA.

**Figure S7.** Most significant up- and down-regulated proteins in 231BR (N = 3 biological triplicates). * denotes the p-value between the two braced cell lines is less than 0.05; ** denotes the p-value less than 0.01; *** denotes the p-value less than 0.001.

**Figure S8.** Unsupervised PCA plots of quantitative proteomes from different cell lines.

**Figure S9**. Matching of significant protein expression changes (p < 0.01, N = 3 biological triplicates) to transcript expression changes in 231 *vs.* 231BR. The color coding represents the fold changes of 231/231BR. Green color denotes the up-regulation in 231 and the down-regulation in 231BR. Red color denotes the down-regulation in 231 and the up-regulation in 231BR.

**Figure S10**. Correlation of protein expression changes and gene expression changes in each cell line when compared to 231BR which is designated as the reference cell line. X-axis is the log transformation of protein fold change. Y-axis is the log transformation of corresponding gene fold change. Log value is applied to indicate the up- or down-regulation. Positive value denotes the up-regulation, while negative value denotes the down-regulation relative to 231BR. Each dot represents the average values of protein expression change *vs.* gene expression change. Only proteins or genes that demonstrated significant expression changes (p < 0.01, N = 3 biological triplicates) and identified in 231BR and the cell line compared to were plotted. Blue dot represents protein and corresponding gene that have the same up- or down-regulation pattern. Red dot represents protein and gene that have the opposite up- or down- regulation pattern. Pearson correlation coefficient of protein and gene expression changes is shown on the bottom right corner of each figure.

**Figure S11.** GO enrichment of Molecular and Cellular Functions of proteins that exhibited significant expression changes when comparing each cell line to 231BR.

**Figure S12.** Integrin signaling pathway of proteomic comparison in 231 *vs.* 231BR. Data were analyzed through the use of IPA (QIAGEN Inc., <https://www.qiagenbioinformatics.com/products/ingenuitypathway-analysis>). Green color denotes the down-regulation in 231 and the up-regulation in 231BR. Red color denotes the up-regulation in 231 and the down-regulation in 231BR.

**Figure S13.** Cell-to-cell signaling and interaction network of proteins that exhibited significant expression changes in 231 *vs.* 231BR. ITGAs have interactions with most proteins, suggesting the central role of ITGAs in the network. Data were analyzed through the use of IPA (QIAGEN Inc., <https://www.qiagenbioinformatics.com/products/ingenuitypathway-analysis>). Green color denotes the down-regulation in 231 and the up-regulation in 231BR. Red color denotes the up-regulation in 231and the down-regulation in 231BR.

Peng, *et al*. Figure S1


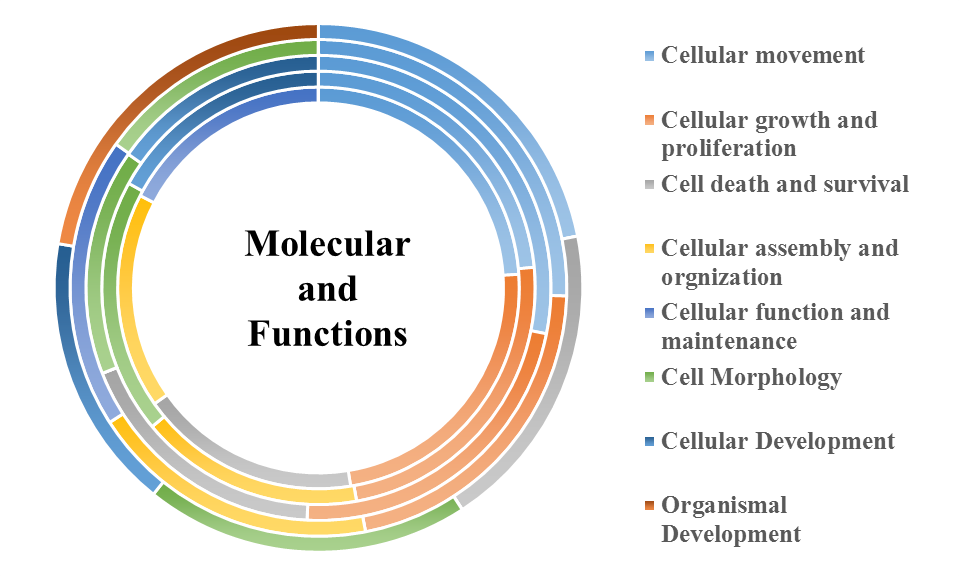


**Figure S1.** Diseases and Biofunctions enrichment of five cell lines compared with 231BR. From inner circle to outer circle are 231, 361, HTB22, HTB131, CRL, respectively, compared with 231BR. The most significant function in all cell lines are cellular movement. The other four functions in top 5 varies among different cell lines. CRL has the biggest difference.

Peng, *et al.* Figure S2


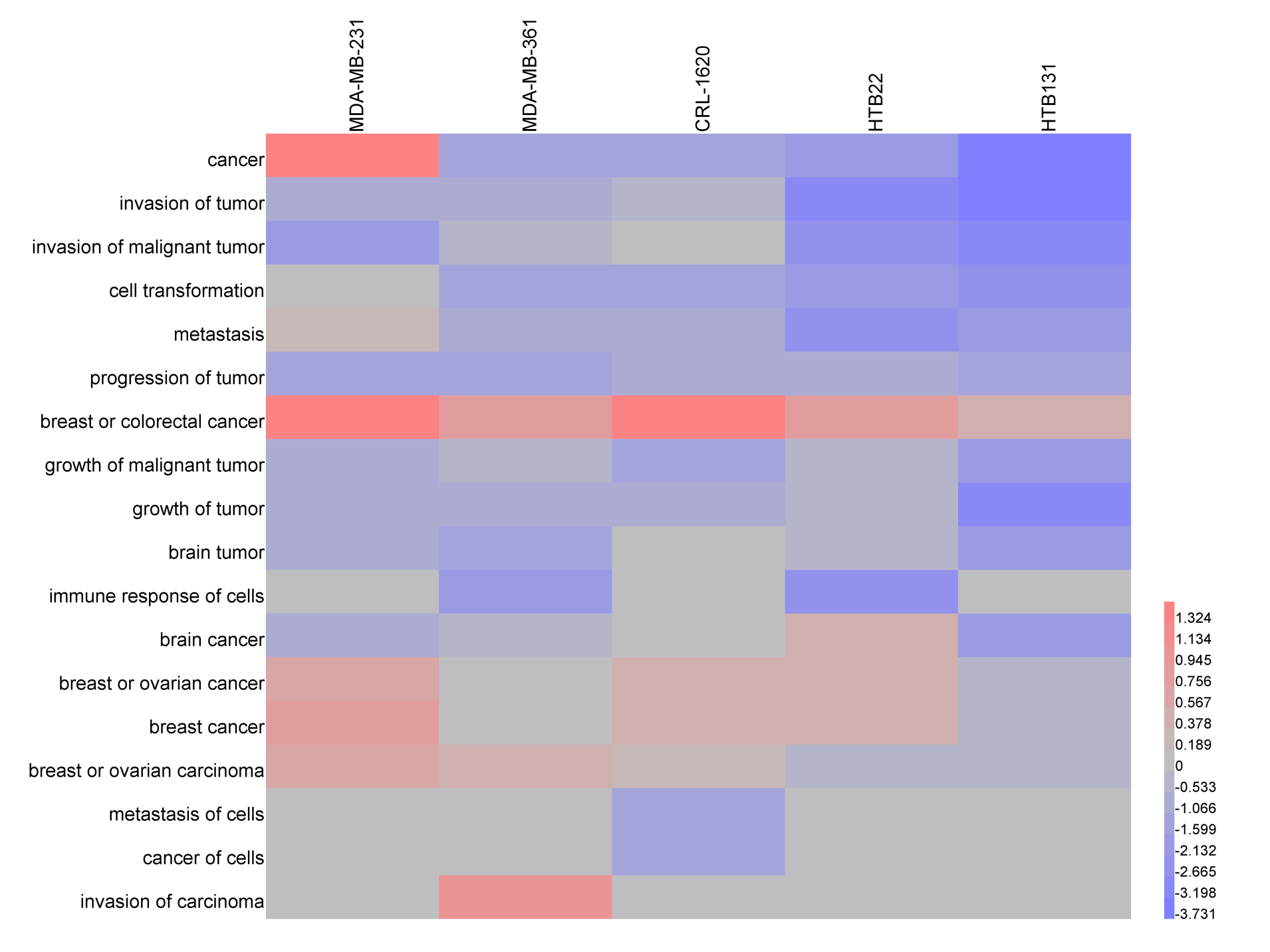


**Figure S2.** Comparison of diseases and disorders in IPA among cell lines. Red color denotes the inhibition of relative functions in each cell line when compared to 231BR and activation in 231BR, and blue color denotes the inhibition in each cell lines and the activation of relative functions in 231BR.

Peng, *et al.* Figure S3

**Figure S3.** VEGF signaling pathway. Green color denotes the down-regulation in 231 compared to 231BR and the up-regulation in 231BR. Red color denotes the up-regulation in 231 and the down-regulation in 231BR. The inserted table depicts the essential expression changes of VEGF genes (N = 3 biological triplicates).

Peng, *et al.* Figure S4


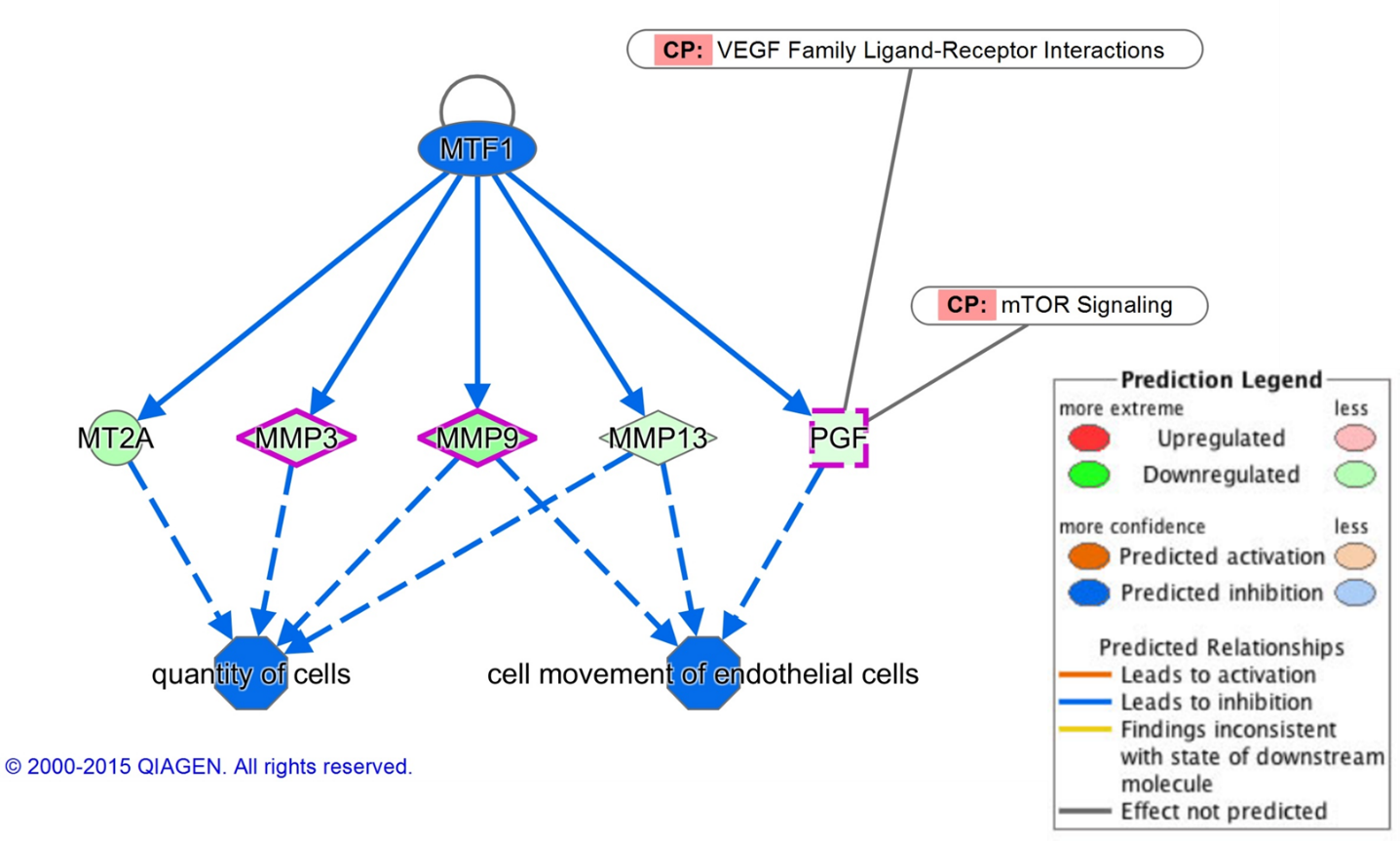


**Figure S4.** Regulator effect analysis in 231 *vs.* 231BR. Green color denotes the down-regulation of genes in 231 and up-regulation in 231BR. Blue color denotes the inhibition of the gene expressions or functions in 231 and activation in 231BR.

Peng, *et al*. Figure S5

**
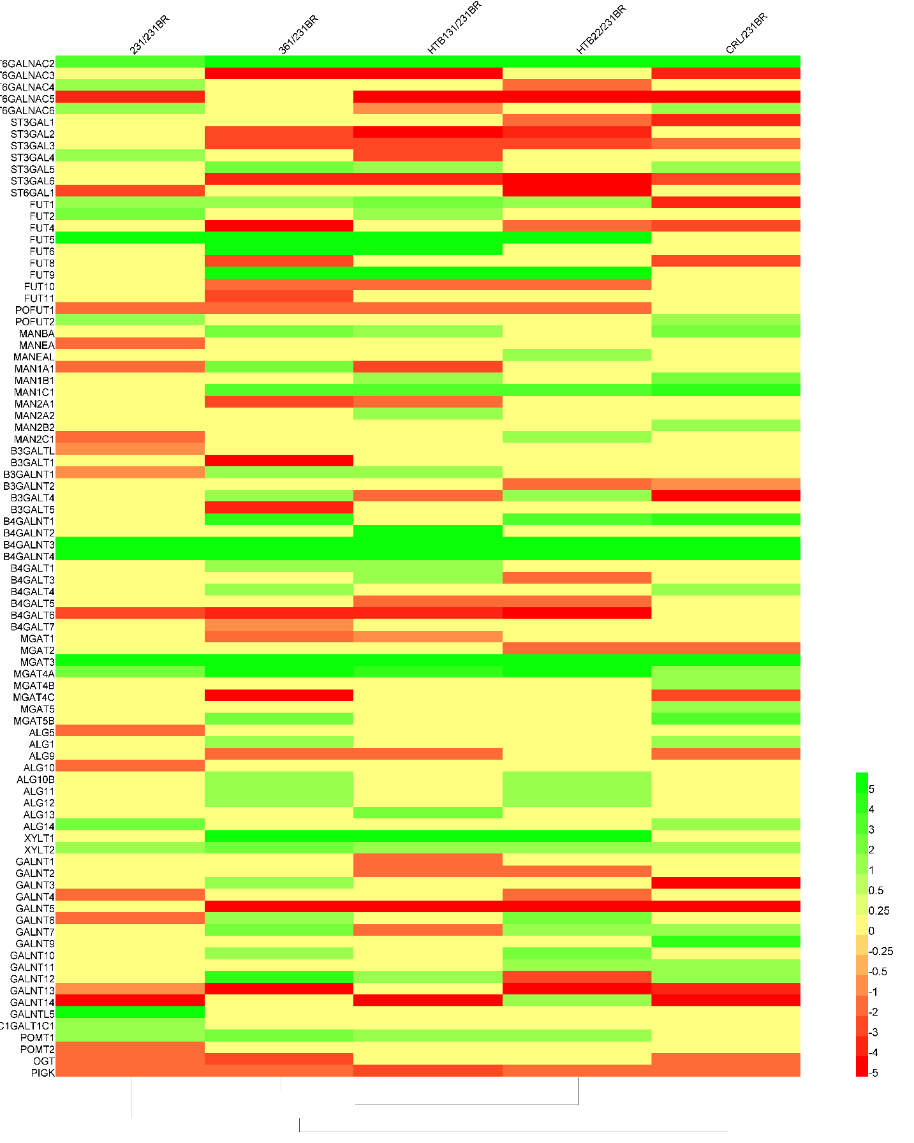
**

**Figure S5.** Glycosylation gene differential expression pattern. Color coding represents the fold changes of genes expressions (RPKM) in other cell lines compared to 231BR. Green color denotes the up-regulation in other cell lines (down-regulation in 231BR). Red color denotes the down-regulation in other cell lines (up-regulation in 231BR).

Peng, *et al.* Figure S6

**A**


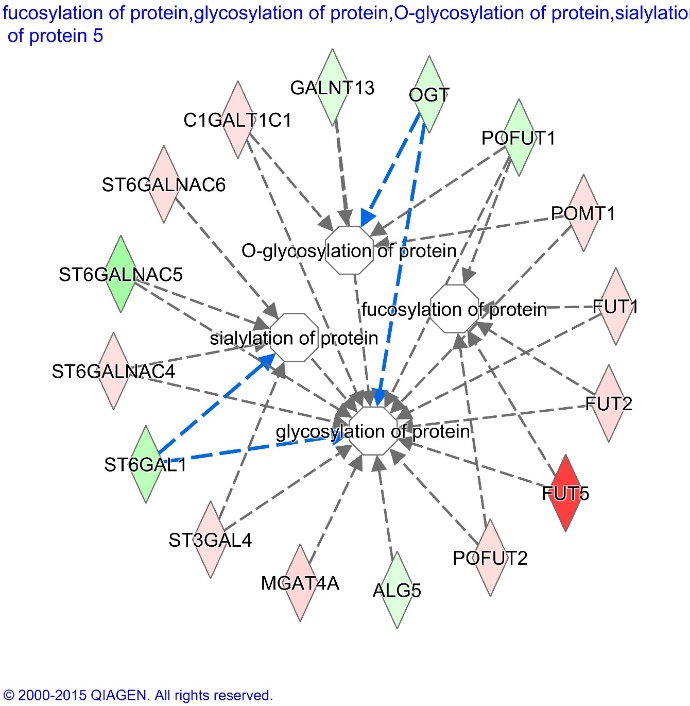


**B**

**
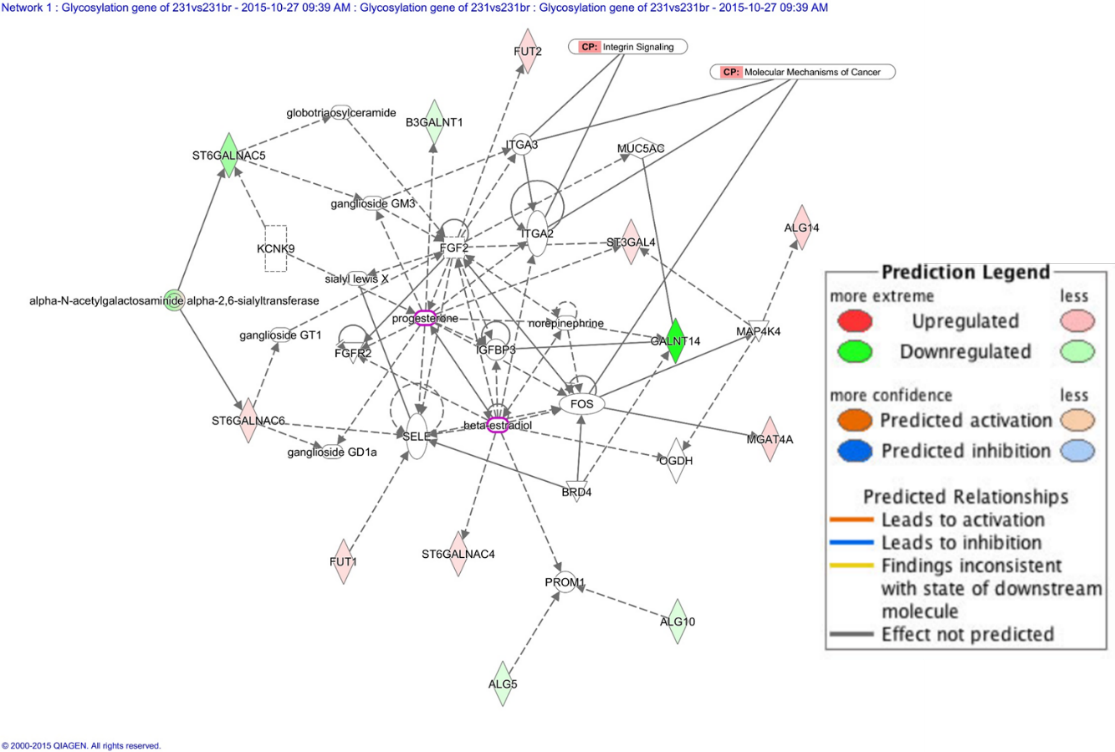
**

**Figure S6.** IPA of glycosylation genes. **A**. functional annotation of glycosylation genes; **B**. glycosylation gene network. Green color denotes the down-regulation of genes in 231 when compared to 231BR and the up-regulation in 231BR. Blue color denotes that the inhibition of the function in 231 and the activation in 231BR. Blue line denotes that the corresponding gene change cause an inhibition to the function. Black line denotes that the effect of gene change cannot be predicted to the function by IPA.

Peng, *et al.* Figure S7


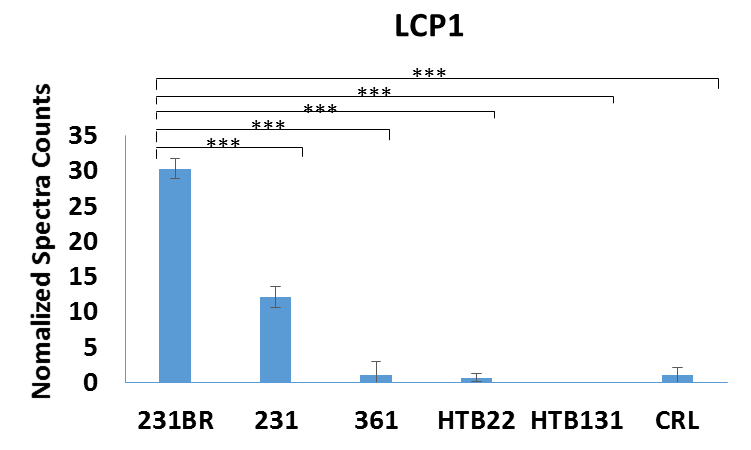

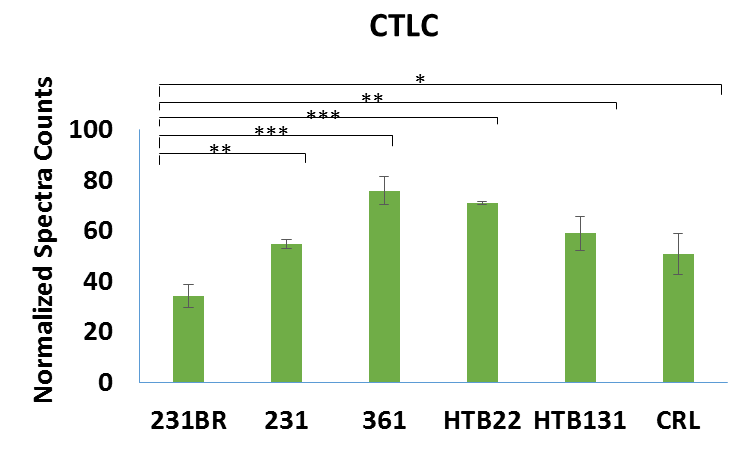


**Figure S7.** Most significant up- and down-regulated proteins in 231BR (N = 3 biological triplicates). * denotes the p-value between the two braced cell lines is less than 0.05; ** denotes the p-value less than 0.01; *** denotes the p-value less than 0.001.

Peng, *et al.* Figure S8


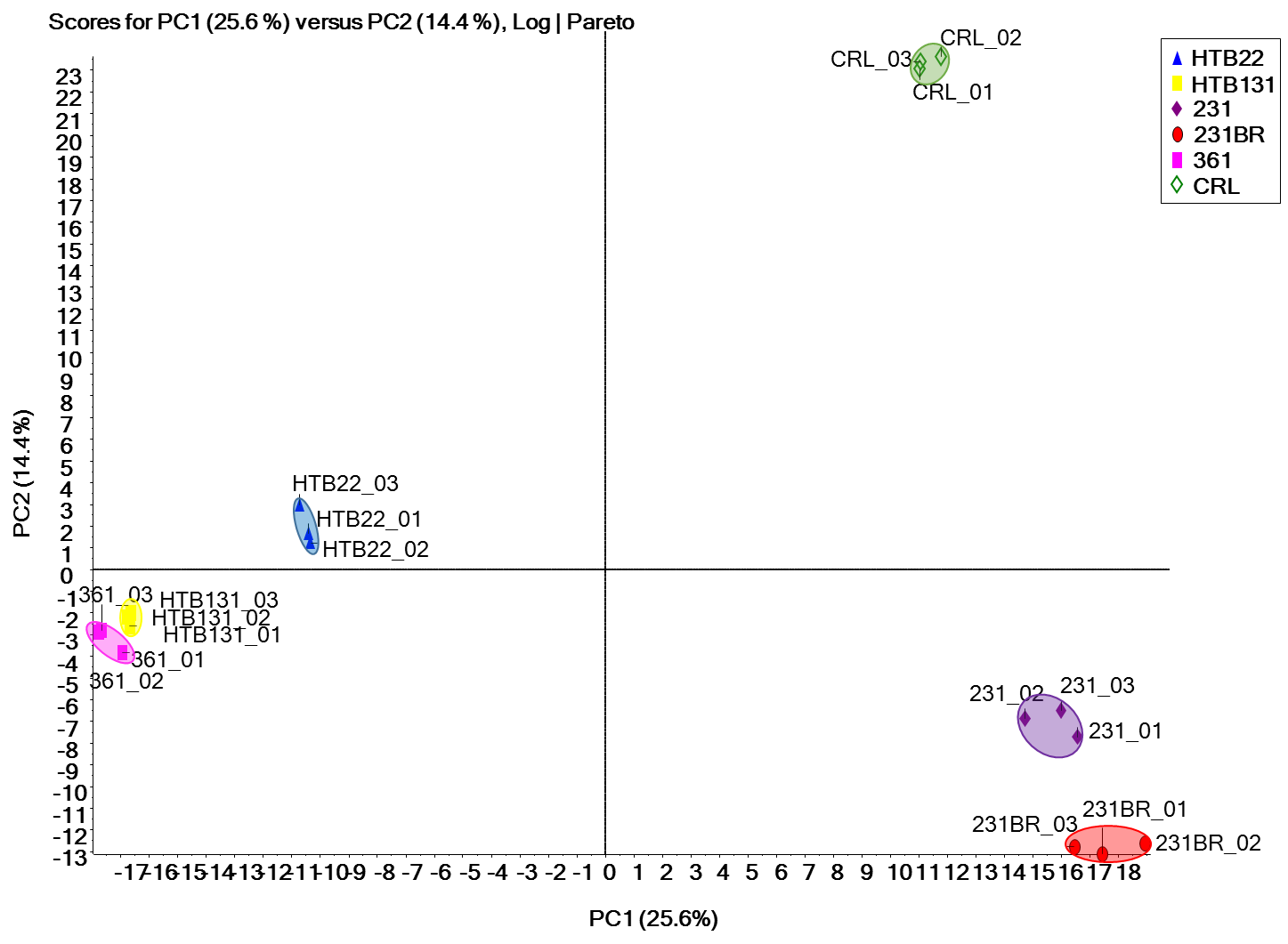


**Figure S8**. Unsupervised PCA plots of quantitative proteomes from different cell lines.

Peng, *et al*. Figure S9


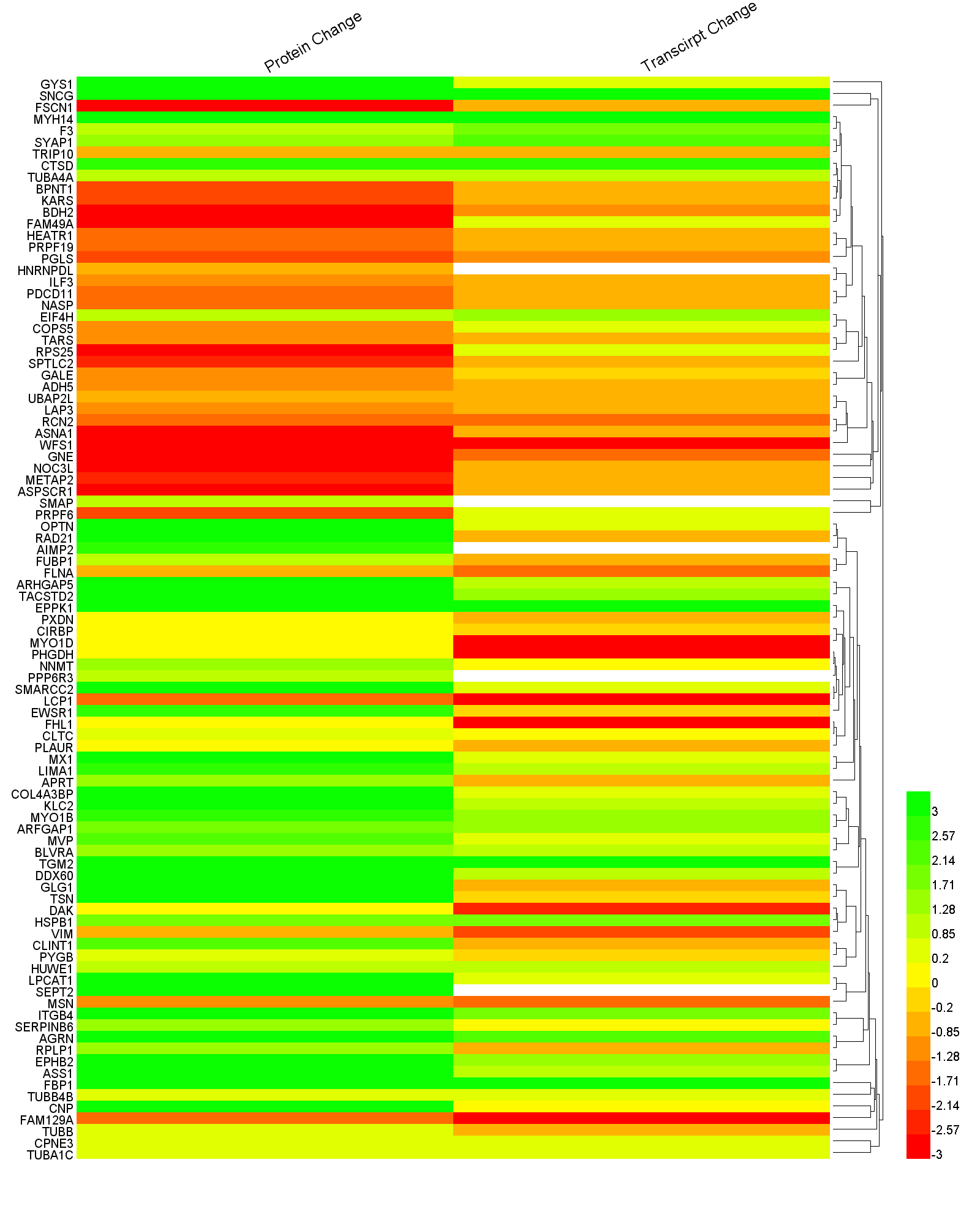


**Figure S9**. Matching of significant protein expression changes (p < 0.01, N = 3 biological triplicates) to transcript expression changes in 231 *vs.* 231BR. The color coding represents the fold changes of 231/231BR. Green color denotes the up-regulation in 231 and the down-regulation in 231BR. Red color denotes the down-regulation in 231 and the up-regulation in 231BR.

Peng, *et al*. Figure S10

**Figure S10**. Correlation of protein expression changes and gene expression changes in each cell line when compared to 231BR which is designated as the reference cell line. X-axis is the log transformation of protein fold change. Y-axis is the log transformation of corresponding gene fold change. Log value is applied to indicate the up- or down-regulation. Positive value denotes the up-regulation, while negative value denotes the down-regulation relative to 231BR. Each dot represents the average values of protein expression change *vs.* gene expression change. Only proteins or genes that demonstrated significant expression changes (p < 0.01, N = 3 biological triplicates) and identified in 231BR and the cell line compared to were plotted. Blue dot represents protein and corresponding gene that have the same up- or down-regulation pattern. Red dot represents protein and gene that have the opposite up- or down- regulation pattern. Pearson correlation coefficient of protein and gene expression changes is shown on the bottom right corner of each figure.

Peng, *et al.* Figure S11

**Figure S11.** GO enrichment of Molecular and Cellular Functions of proteins that exhibited significant expression changes when comparing each cell line to 231BR.

Peng, *et al.* Figure S12


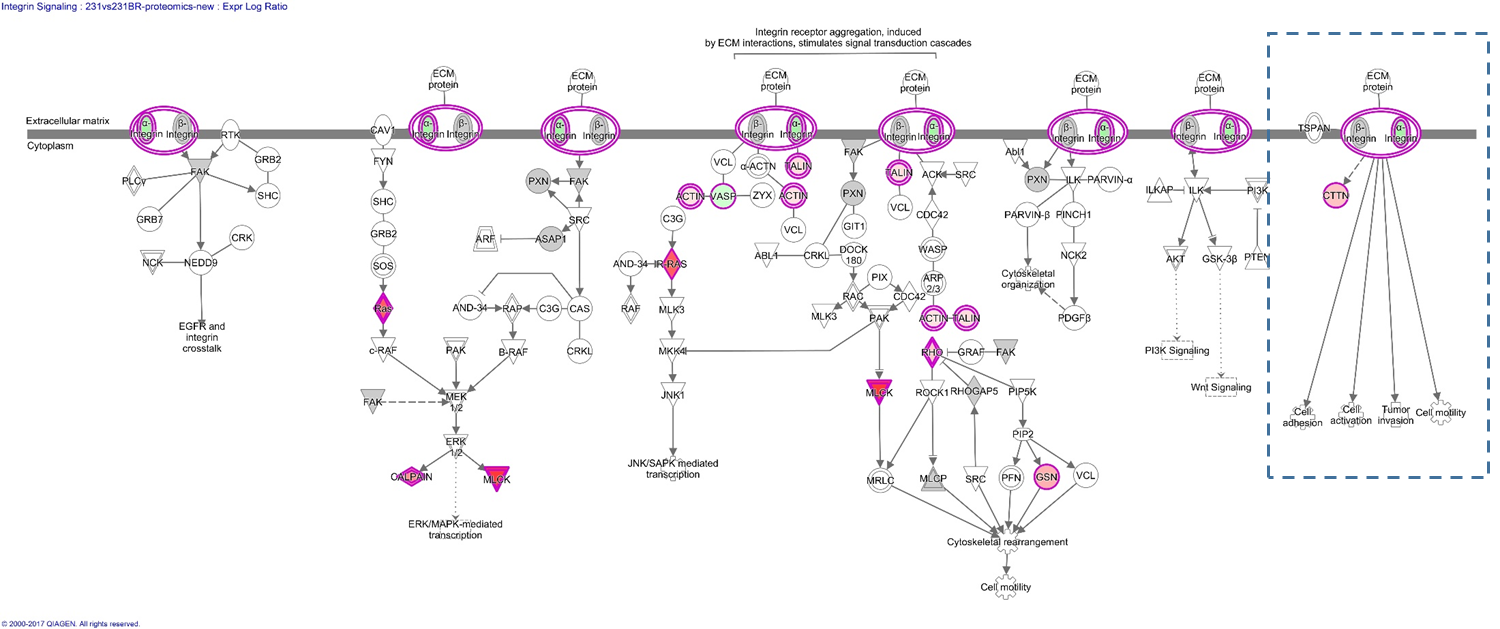


**Figure S12.** Integrin signaling pathway of proteomic comparison in 231 *vs.* 231BR. Green color denotes the down-regulation in 231 and the up-regulation in 231BR. Red color denotes the up-regulation in 231 and the down-regulation in 231BR.

Peng, *et al.* Figure S13


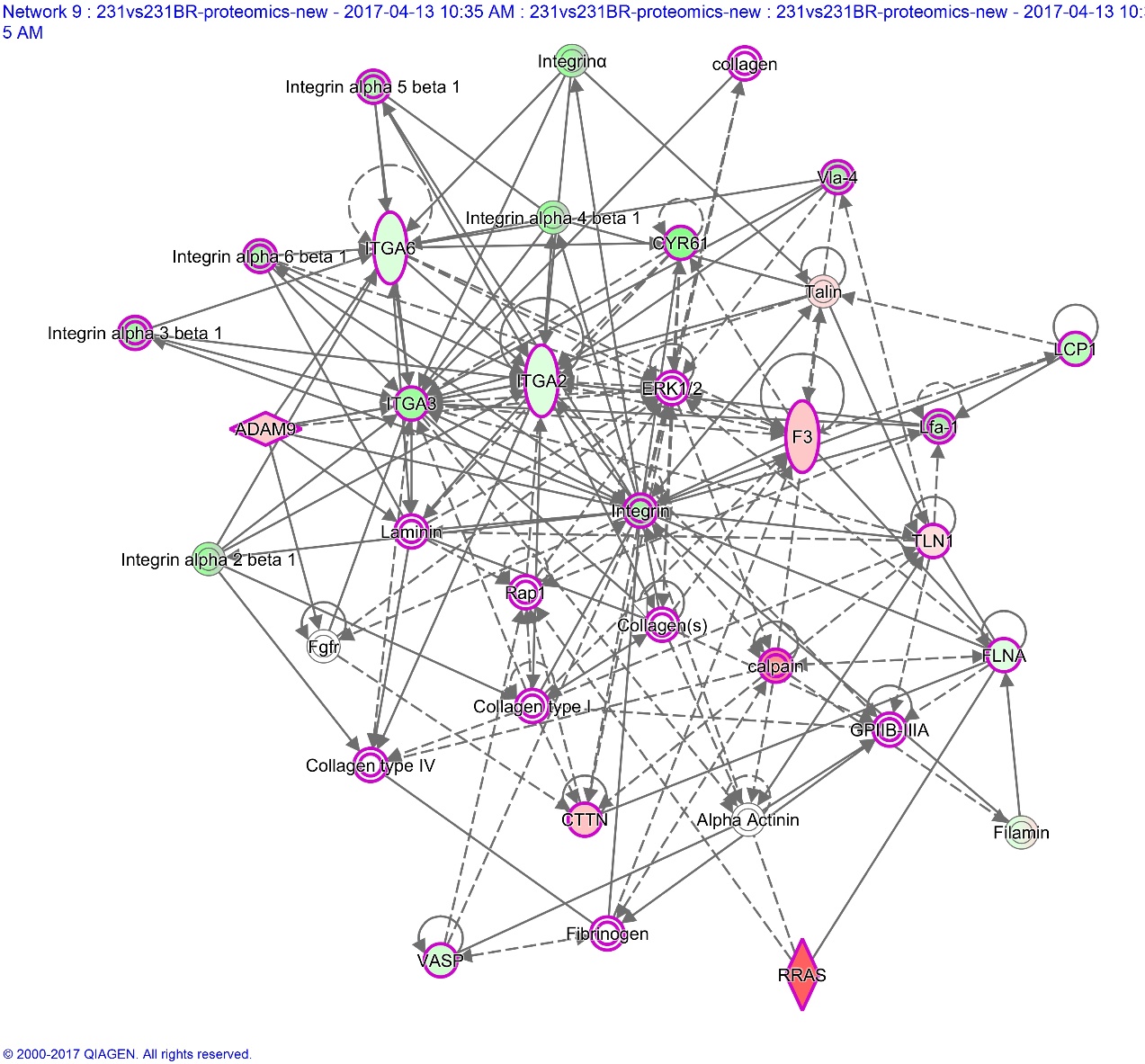


**Figure S13.** Cell-to-cell signaling and interaction network of proteins that exhibited significant expression changes in 231 *vs.* 231BR. ITGAs have interactions with most proteins, suggesting the central role of ITGAs in the network. Green color denotes the down-regulation in 231 and the up-regulation in 231BR. Red color denotes the up-regulation in 231and the down-regulation in 231BR.
